# Adaptive control for shape memory alloy actuated systems with applications to human–robot interaction

**DOI:** 10.3389/fnins.2024.1337580

**Published:** 2024-01-31

**Authors:** Enming Shi, Xu Zhong, Tian Wang, Xiaoguang Li, Chunguang Bu, Xingang Zhao

**Affiliations:** ^1^State Key Laboratory of Robotics, Shenyang Institute of Automation, Chinese Academy of Sciences, Shenyang, Liaoning, China,; ^2^Institutes for Robotics and Intelligent Manufacturing, Chinese Academy of Sciences, Shenyang, Liaoning, China; ^3^University of Chinese Academy of Sciences, Beijing, China; ^4^Medical Engineering Department, Affiliated Hospital of Yangzhou University, Yangzhou, Jiangsu, China; ^5^The Fourth Affiliated Hospital of China Medical University, Shenyang, China; ^6^School of Intelligent Manufacturing, Huzhou College, Zhejiang, China

**Keywords:** adaptive control, SMA actuator, gray-box model, robustness, hand rehabilitation robots

## Abstract

**Introduction:**

Shape memory alloy (SMA) actuators are attractive options for robotic applications due to their salient features. So far, achieving precise control of SMA actuators and applying them to human-robot interaction scenarios remains a challenge.

**Methods:**

This paper proposes a novel approach to deal with the control problem of a SMA actuator. Departing from conventional mechanism models, we attempt to describe this nonlinear plant using a gray-box model, in which only the input current and the output displacement are measured. The control scheme consists of the model parameters updating and the control law calculation. The adaptation algorithm is founded on the multi-innovation concept and incorporates a dead-zone weighted factor, aiming to concurrently reduce computational complexities and enhance robustness properties. The control law is based on a PI controller, the gains of which are designed by the pole assignment technique. Theoretical analysis proves that the closed-loop performance can be ensured under mild conditions.

**Results:**

The experiments are first conducted through the Beckhoff controller. The comparative results suggest that the proposed adaptive PI control strategy exhibits broad applicability, particularly under load variations. Subsequently, the SMA actuator is designed and incorporated into the hand rehabilitation robot. System position tracking experiments and passive rehabilitation training experiments for various gestures are then conducted. The experimental outcomes demonstrate that the hand rehabilitation robot, utilizing the SMA actuator, achieves higher position tracking accuracy and a more stable system under the adaptive control strategy proposed in this paper. Simultaneously, it successfully accommodates hand rehabilitation movements for multiple gestures.

**Discussion:**

The adaptive controller proposed in this paper takes into account both the computational complexity of the model and the accuracy of the control results, Experimental results not only demonstrate the practicality and reliability of the controller but also attest to its potential application in human-machine interaction within the field of neural rehabilitation.

## 1 Introduction

Shape memory alloy (SMA) actuators present numerous advantageous features, including excellent volume output ratios, low driving voltages, and noiseless and clean actuations (Shi et al., 2017). These attributes render SMA actuators appealing choices for rehabilitation robotic systems. The inherent shape memory effects of SMAs allow them to revert to predefined shapes upon proper heating. Nevertheless, these effects introduce nonlinearities, parameter uncertainties, and hysteresis into the control problem (Wiest and Buckner, 2014). As of now, achieving precise control of SMA actuators remains an unresolved and highly relevant challenge, serving as the primary motivation for this study.

In the literature, a particularly intuitive approach involves the design of controllers based on mechanism models. Romano and Tannuri (2009) exemplified this approach by creating a mechanical actuator using SMA. The mechanism model, derived from experimental setups, encompasses a thermal model, a phase transformation model, and a description of the mechanical properties and dynamics of the system. Elahinia and Ashrafiuon (2002) developed a sliding mode control (SMC) method based on a mechanism model. Given that this control law necessitates full state feedback, the extended Kalman filter is employed to update the unmeasurable states. In Ashrafiuon and Jala (2009), this approach was implemented in a three-degrees-of-freedom robotic manipulator. Zakerzadeh and Sayyaadi (2013) investigated hysteresis behaviors and integrated a feed-forward controller into an adaptive controller, relying on the inverse of the hysteresis model. Riccardi et al. (2013) addressed magnetic SMA actuators, introducing a novel technique to compensate for hysteresis nonlinearity. In a related context, Pai et al. (2017) proposed a force control strategy grounded in the mechanism model. Despite the contributions, mechanism model-based controllers have limitations: (a) the structures of mechanism models are often very complex; (b) updating model parameters recursively is quite challenging, and they typically remain fixed for online implementation; (c) the inverse hysteresis models are also very intricate and lack adaptability.

As an alternative, neural network models have attracted attention due to their approximation accuracy and structural flexibility. Tai and Ahn (2010) introduced a model for an SMA actuator based on radial basis function neural networks, with parameters updated through online learning. Nikdel et al. (2014) compared the neural model predictive control method with the SMC approach. In a related context, Son and Anh (2015) proposed an adaptive feed-forward neural network model to compensate for hysteresis nonlinearity. The model proposed by Son and Anh (2015) is constructed by integrating multi-layer perceptron neural networks with a linear model. Tai and Ahn (2012) combined the advantages of a direct adaptive controller with neural network approximations, showcasing effectiveness in compensating for hysteresis and ensuring reliable robustness. In a related context, Wiest and Buckner (2014) tackled antagonistic SMA systems using a hysteretic recurrent neural network. Meanwhile, Pan et al. (2017) focused on a novel SMA actuator designed with reduced total stiffness and increased compliance. Neural networks are utilized to model this nonlinear plant. The effectiveness of the adaptive observer-based output-feedback controller in handling load changes is demonstrated. However, despite these merits, several key issues still need to be addressed: (a) online training of neural networks may face challenges associated with local minima; (b) conducting robust stability analysis for systems based on neural networks has proven to be difficult; (c) controllers based on neural networks often involve significant computational complexities and may be impractical for specific applications.

On the other hand, the pseudoelasticity and shape memory effect (SME) of SMA hold significant application value in neurology and neuromuscular rehabilitation applications (Pittaccio et al., 2015). Specifically, pseudoelasticity has been proposed in various studies, including limb positioning and gait rehabilitation (Viscuso et al., 2009; Deberg et al., 2014; Mataee et al., 2015). In these studies, the adaptability, deformability, and nonlinear mechanical properties of SMA are considered effective in addressing clinical issues associated with spasticity and paralysis. Similarly, SME can provide the foundational characteristics for the design of neural rehabilitation devices, including quasi-constant stress levels and a larger range of deformation, and these parameters can be manipulated through thermomechanical processing for structural design and repair (Pittaccio et al., 2015). SME also enables the SMA to integrate the sensor with actuator which can simplify the structure (Wang et al., 2021). In addition, SMA actuators are frictionless, quiet, corrosion-resistant, offer an extended fatigue life, and demonstrate high damping and resistivity (Kumbhar et al., 2017; Shariat et al., 2017). These characteristics reduce actuator complexity, size, and weight. Therefore, several research teams have employed SMA in wearable rehabilitation devices and have devised corresponding system control algorithms. Serrano et al. (2018) introduced an SMA-actuated wrist-based exoskeleton with a lightweight and comfortable design. Additionally, Serrano et al. (2023) developed a flexible exo-glove powered by SMA, capable of executing complex gestures. Jeong et al. proposed a wrist exoskeleton robot driven by SMA springs, featuring a high contraction strain capacity. However, its coil structure is complex, and despite the establishment of a complicated thermodynamic model, the accuracy of the model remains unsatisfactory (Jeong et al., 2019, 2022). Wang et al. (2021) presented a flexible hand motion device powered by SMA wires. This device controls the angle of the robot finger joints by adjusting the duty cycle of the PWM pulses. However, the study does not delve into the robustness considerations of the robot system. Xie et al. (2023) embedded SMA into a conformal material and proposed a hand rehabilitation wearable glove actuated by an SMA-based Soft Composite Structure (SSCS). This structure is characterized by simple actuation and a large force-to-weight ratio. However, its precision in the motion control of the hand is noted to be imprecise. Lai et al. (2023) introduced a hybrid actuator combining a flexible actuator and an SMA spring actuator, integrated into a soft glove. This configuration offers a larger workspace and enhanced output force. However, there is potential for improvement in the tracking accuracy of the control system and the anti-interference capability. Considering the above set of research results, it is clear that ensuring model simplicity and improving the accuracy of control results are extremely challenging issues. They directly affect the overall control effectiveness and practicality of the actuator.

To this end, this paper proposes describing a SMA actuator using a gray-box model. This simple model comprises a first-order discrete linear model and unmodeled dynamics, leveraging measurements of the actuator’s input current and output displacement as data-driven components. Only two model parameters are updated online, resulting in a low computational burden. To enhance system robustness and reject disturbances, a novel identification algorithm with a dead-zone weighted factor is introduced. Robust estimation of unmodeled dynamics is necessary, as it can be directly compensated by an adaptive control law. In line with the adaptations, the proportional and differential gains of the PI control law are updated online based on a pre-specified stable characteristic polynomial. The overall adaptive control algorithms are explicit and have been successfully implemented in both the Beckhoff controller and the embedded system. More interestingly, this method proves to be applicable for handling load variations and rejecting disturbances. Furthermore, the integration of the SMA actuator into the hand rehabilitation robot system allows for position tracking experiments and hand rehabilitation training. These experiments are conducted based on the data-driven modeling method and the robust adaptive control strategy proposed in this paper. The most important contribution of this paper is that, oriented to the SMA actuator, a comprehensive method of control system design is proposed, which takes into account both the model computational complexity problem and the control accuracy problem, tries to give a more reasonable solution, and makes this adaptive control technology effectively applied in the rehabilitation robot system.

This paper is organized as follows: the problem formulation and the adaptive controller are proposed in Section 2, the experiments and results are presented in Section 3, a brief summary is given in Section 4, and the closed-loop stability is analyzed in Appendix.

## 2 Methods

### 2.1 SMA characteristics

**Figure 1:**
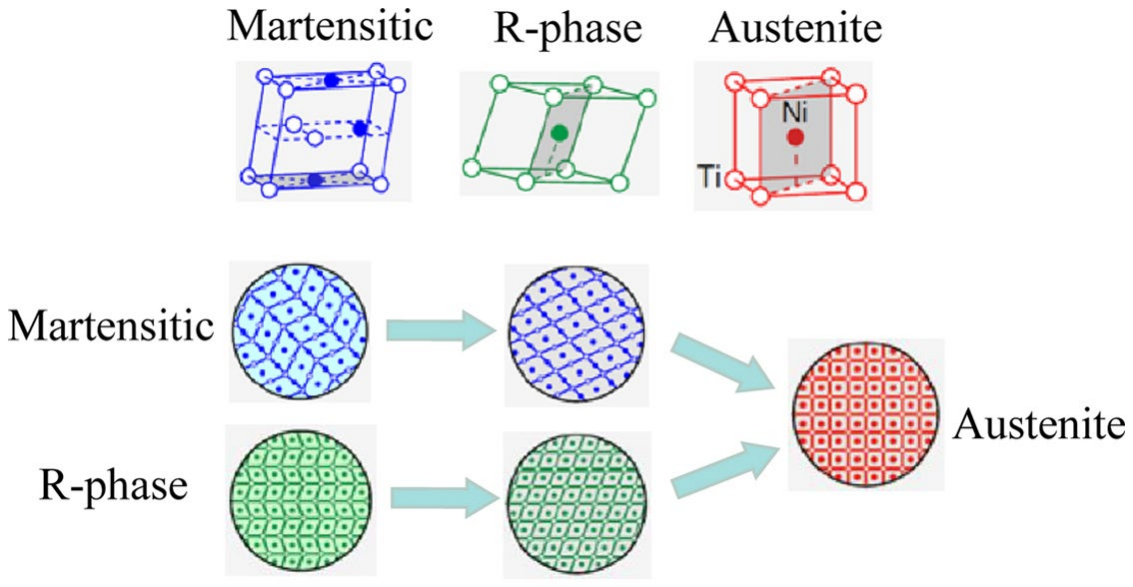
Microstructural changes in shape memory alloys.

The SME of SMA wires refers to the fact that the unconstrained deformed alloy wire material can be restored to its original shape under the condition of external temperature change (Airoldi et al., 1991). On a microscopic level, the shape memory properties of a SMA wire are caused by changes in its own structure. SMA wires have two main crystal states, a martensitic phase at low temperatures, when the SMA wires have a monoclinic crystal shape inside; The other is the austenite phase at high ambient temperatures, when the material exhibits a cubic crystal structure internally; In addition to these two states, SMA wires also have an R-phase state at intermediate temperatures, when the material has an internal monoclinic crystal structure. The essence of SME is the migration of highly ordered “militarization” of crystal atoms within the SMA wire (Lagoudas and Dimitris, 2008), from monoclinic to cubic crystal structure, and the deformation of the SMA wire is achieved by the change of countless such microcrystal structures, a process known as the martensitic phase transition, as shown in Figure 1.

**Figure 2:**
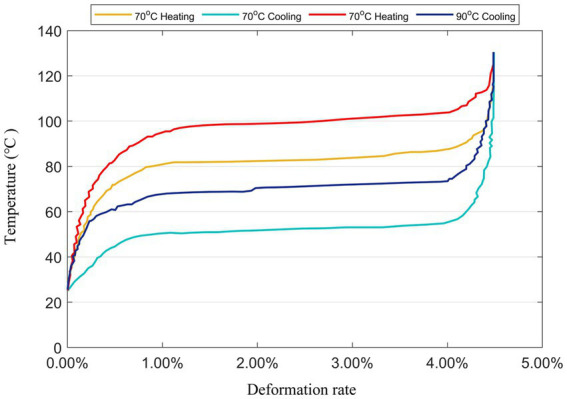
Deformation rate of SMA at different phase transition temperatures.

There are many types of constituent materials of shape memory alloy wires, and the different properties of different materials lead to differences in the shape memory function of SMAs, and researchers have categorized the SME into three types according to the differences in the shape memory function: the single-pass memory effect, the two-pass memory effect, and the whole-pass memory effect (Wu et al., 1996). The SMA wires used in this study were dual-range memory effect SMA wires. Dual-range SMA wires have a shape memory effect when they are deformed and processed, and they change back to their original shape when heated to a certain temperature, and then regain their length when cooled. Different heat treatments during processing also have a great influence on the SMA wires. Figure 2 shows the deformation and temperature curves of SMA wires selected with the same diameter and phase transition temperatures of 70 and 90°C, respectively, in the process of heating and cooling. As depicted in the figure, it is evident that the temperature of deformation increases with the higher temperature of the heat treatment.

### 2.2 Gray-box model description

Plenty of research has demonstrated a fact that it is almost impossible to precisely capture the nonlinear dynamics of SMA actuators during a relatively wide range. An alternative idea to address the unmodeled dynamics is to compensate the negative effects in the subsequent control problem, rather than copy with it directly in the modeling problem.

In other words, for the modeling aspect of this work, the aim is to approximately capture the main dynamic property of this actuator, based on a computationally efficient model. Later, the adaptive controller will ensure the robust stability despite the unmodeled dynamics.

Let the single-input-single-output SMA actuator be described as a discrete-time nonlinear dynamical system in the following form:


(1) $$y\left(t+1\right)=\phi \left[y\left(t\right),\ldots ,y\left(t+1-n_{a}\right),u\left(t\right),\ldots ,u\left(t+1-n_{b}\right)\right]$$

where the system output *y*(*t*) is the displacement of the SMA wire (unit: *m*); the system input *u*(*t*) is the current signal (unit: *A*); *n_a_* and *n_b_* are unknown system orders; *ϕ*[⋅] is a nonlinear function. The origin can be assumed as an equilibrium point.

By using Taylor’s formula around the origin, the nonlinear system (Equation 1) can be equivalently expressed as a first-order linear model together with unmodeled dynamics:


(2) $$A\left(z^{-1}\right)y\left(t\right)=B\left(z^{-1}\right)u\left(t\right)+\zeta \left(t\right)$$

where $A\left(z^{-1}\right)$ and $B\left(z^{-1}\right)$ are polynomials in the time delay operator $z^{-1}\left\{e.g.z^{-1}u\left(t\right)=u\left(t-1\right)\right\}$ which are defined as:


$$A\left(z^{-1}\right)=1+a_{1}z^{-1}$$

$$B\left(z^{-1}\right)=b_{1}z^{-1}$$

where *a*_1_ and *b*_1_ are the uncertain system parameters; ζ(*t*) is the unmodeled dynamics, which is unknown and varies due to temperature changes, load variations or other factors.

The system Equation (2) can be written as a compact form


(3) $$y\left(t\right)=\varphi^{T}\left(t\right)\theta +\zeta \left(t\right)$$

where the parameter vector θ and the regressor vector φ(*t*) are defined as follows:


(4) $$\theta ={\left[a_{1},b_{1}\right]}^{T}$$

(5) $$\varphi \left(t\right)={\left[-y\left(t-1\right),u\left(t-1\right)\right]}^{T}$$

Regardless of ζ(*t*), the prediction model is considered as:


(6) $$y\left(t+1\right)≜\varphi^{T}\left(t+1\right)\hat{\theta }\left(t\right)$$

with $\hat{\theta }\left(t\right)$ defined as the estimation of θ:


(7) $$\hat{\theta }\left(t\right)={\left[{\hat{a}}_{1}\left(t\right),{\hat{b}}_{1}\left(t\right)\right]}^{T}$$

We can approximately capture the main dynamic property of the nonlinear plant by the discrete linear model (Equation 6). Inevitably, there exist modeling errors based on this simple model. But it will be proved in the Appendix that the unmodeled dynamics can be compensated by the proposed PI controller.

### 2.3 An adaptive control strategy

The utilized PI controller is written as:


(8) $$u\left(t\right)=u\left(t-1\right)+k_{P}\left[\varepsilon \left(t\right)-\varepsilon \left(t-1\right)\right]+k_{I}\varepsilon \left(t\right)$$

where *k_p_* and *k_I_* are the proportional and integral gains, and $\varepsilon \left(t\right)=y^{*}\left(t\right)-y\left(t\right)$ with $y^{*}\left(t\right)$ defined as the reference.

**Figure 3:**
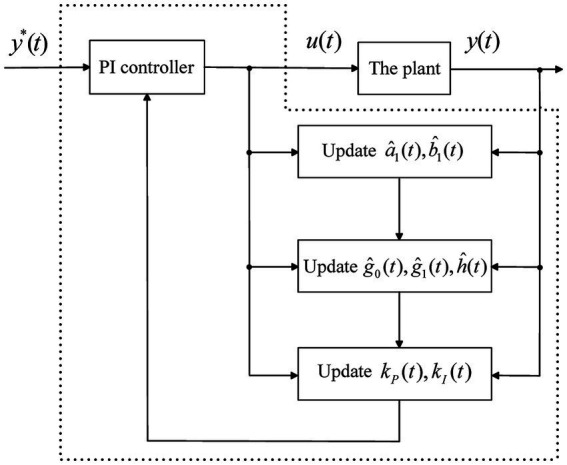
The adaptive PI control scheme.

It is desired that the system output tracks the reference, and the robust stability is ensured under uncertainties. The adaptive PI control scheme is briefly depicted in Figure 3, which consists of online parameter adaptation and control law calculation.

#### 2.3.1 Control law for deterministic systems

The above control law can be written in the following form:


(9) $$H\left(z^{-1}\right)u\left(t\right)=G\left(z^{-1}\right)\varepsilon \left(t\right)$$

where $H\left(z^{-1}\right)=h\left(1-z^{-1}\right)$ and $G\left(z^{-1}\right)=g_{0}+g_{1}z^{-1}$ . Note that the proportional and differential gains in Equation (8) are chosen by the following relation Equation (10).


(10) $$\left\{ \begin{array}{c} k_{P}+k_{I}=\frac{g_{0}}{h}\\ -k_{P}=\frac{g_{1}}{h} \end{array}\right.$$

An effective technique to design the polynomials $H\left(z^{-1}\right)$ and $G\left(z^{-1}\right)$ is based on the pole assignment concept (Goodwin and Sin, 1984).

Applying the controller Equation (8) and combining Equation (3) with Equation (9) yield the closed-loop Equation (11).


(11) $$\begin{array}{l} \left[A\left(z^{-1}\right)H\left(z^{-1}\right)+B\left(z^{-1}\right)G\left(z^{-1}\right)\right]y\left(t+1\right)\\ \;=B\left(z^{-1}\right)G\left(z^{-1}\right)y^{*}\left(t\right)+H\left(z^{-1}\right)\zeta \left(t\right) \end{array}$$

Let the closed-loop characteristic polynomial be defined as $T\left(z^{-1}\right)=t_{0}+t_{1}z^{-1}+t_{2}z^{-2}$ , which has stable poles.

When $A\left(z^{-1}\right)$ and $B\left(z^{-1}\right)$ are completely known, to ensure the closed-loop stability, the polynomials $H\left(z^{-1}\right)$ and $G\left(z^{-1}\right)$ should be designed based on:


(12) $$T\left(z^{-1}\right)=A\left(z^{-1}\right)H\left(z^{-1}\right)+B\left(z^{-1}\right)G\left(z^{-1}\right)$$

From Equations (2), (9), and (12), the coefficients are:


(13) $$h=t_{0},g_{0}=\frac{t_{1}+t_{0}-a_{1}t_{0}}{b_{1}},g_{1}=\frac{t_{2}+a_{1}t_{0}}{b_{1}}$$

Further based on Equation (10), the proportional and differential gains *k_p_* and *k_I_* are designed as follows:


(14) $$k_{P}=-\frac{t_{2}+a_{1}t_{0}}{b_{1}t_{0}},k_{I}=\frac{t_{1}+t_{2}+t_{0}}{b_{1}t_{0}}$$

The above analysis is carried out based on the deterministic model. However, such an assumption is unrealistic for the SMA actuator. Actually, the parameters *a*_1_ and *b*_1_ of the gray-box model are uncertain, and it is difficult to offline choose fixed and appropriate *k_p_* and *k_I_* to ensure the closed-loop stability during the whole operating range. A more reasonable treatment seems to estimate $A\left(z^{-1}\right)$ and $B\left(z^{-1}\right)$ recursively, to update $H\left(z^{-1}\right)$ and $G\left(z^{-1}\right)$ online, and then to calculate *k_p_* and *k_I_*.

#### 2.3.2 Online adaptation algorithm

This subsection presents an online adaptation algorithm for uncertain parameters. Recursive least squares (RLS) algorithm has a fast convergence rate. However, it has high computational complexities, especially when it is applied to the Beckhoff IPC programming. On the other hand, recursive stochastic gradient (RSG) algorithm is more favorable to model adaptations, but it leads to a much slower convergence rate. To this end, a novel recursive estimator will be introduced, which has a similar form as the RSG algorithm, but possesses a similar convergence rate as the RLS algorithm.

The parameter identification will be carried out based on Equation (3). We first impose an assumption on this system.

Assumption 1: The unmodeled dynamics ζ(*t*) satisfies


(15) $$\left|\zeta \left(t\right)\right|\leq \Delta$$

where the bound Δ is user-designed.

Remark 1: This condition is commonly used to improve the robustness performance (Goodwin and Sin, 1984). The unmodeled dynamics can be treated as a bounded disturbance, and the parameter estimation can reject some continuous perturbations. The bound Δ is easy to design according to the control performance.

Then the uncertain parameter estimation vector $\hat{\theta }\left(t\right)$ can be updated by the following modified recursive multi-innovation stochastic gradient identification algorithm (Zhang et al., 2008) with a novel dead-zone weighted factor:


(16) $$E\left(t\right)={\left[e\left(t\right),e\left(t-1\right),\ldots ,e\left(t-p+1\right)\right]}^{T}$$

(17) $$\Phi \left(t\right)=\left[\varphi \left(t\right),\varphi \left(t-1\right),\ldots ,\varphi \left(t-p+1\right)\right]$$

(18) $$e\left(t\right)=y\left(t\right)-\varphi^{T}\left(t\right)\hat{\theta }\left(t-1\right)$$

(19) $$r\left(t\right)=r\left(t-1\right)+{\left\|\Phi \left(t\right)\right\|}_{2}^{2}$$

(20) $$\lambda \left(t\right)=\{\begin{array}{l} 1-\frac{\sqrt{p}\Delta }{{\left\|E\left(t\right)\right\|}_{2}},\mathrm{if}\;{\left\|E\left(t\right)\right\|}_{2}>\sqrt{p}\Delta \\ 0,\mathrm{otherwise} \end{array}$$

(21) $$\hat{\theta }\left(t\right)=\hat{\theta }\left(t-1\right)+\frac{\varepsilon \lambda \left(t\right)\Phi \left(t\right)E\left(t\right)}{r\left(t\right)}$$

where *p* is the dimension of the extended signals, which is designed by the user; *r*(0)=1; *e*(*t*) is the model error; *E*(*t*) is the extended model error; Φ(*t*) is the extended regressor vector; λ(*t*) is a nonnegative weighted factor; ε is a user-designed adaptation gain and satisfies 0 < ε ≤ 2 (*Lemma 1* will explain the reason).

Remark 2: It is seen that when *p* = 1, the algorithm becomes a RSG one. Ding and Chen (2006) proved that when *p* increases, the convergence rate of a multi-innovation-based identification algorithm tends to an RLS one. To make a tradeoff between the convergence rate and the computational complexities, we will select *p* = 3. More interestingly, the update of each parameter can be separated and written in one dimensional form, such as ${\hat{a}}_{1}\left(t\right)={\hat{a}}_{1}\left(t-1\right)-\left[\sum_{n=0}^{2}y\left(t-n-1\right)e\left(t-n\right)\right]\cdot \varepsilon \lambda \left(t\right)/r\left(t\right)\text{.}$

#### 2.3.3 Control law update

Based on Equation (7), the estimated polynomials at instant *t* can be defined as:


(22) $$\hat{A}\left(t,z^{-1}\right)=1+{\hat{a}}_{1}\left(t\right)z^{-1}$$

(23) $$\hat{B}\left(t,z^{-1}\right)={\hat{b}}_{1}\left(t\right)z^{-1}$$

In order to adaptively design the proportional and differential gains *k_p_* and *k_I_* for the PI control Equation (8), a modified relationship is as follows:


(24) $$\hat{H}\left(t,z^{-1}\right)u\left(t\right)=\hat{G}\left(t,z^{-1}\right)\varepsilon \left(t\right)$$

where $\hat{H}\left(t,z^{-1}\right)$ and $\hat{G}\left(t,z^{-1}\right)$ are used in place of $H\left(z^{-1}\right)$ and $G\left(z^{-1}\right)$ . These two polynomials are defined as:


(25) $$\hat{H}\left(t,z^{-1}\right)=\hat{h}\left(t\right)\left(1-z^{-1}\right)$$

(26) $$\hat{G}\left(t,z^{-1}\right)={\hat{g}}_{0}\left(t\right)+{\hat{g}}_{1}\left(t\right)z^{-1}$$

It is desired that the polynomials $\hat{H}\left(t,z^{-1}\right)$ and $\hat{G}\left(t,z^{-1}\right)$ satisfy the following relation:


(27) $$T\left(z^{-1}\right)=\hat{A}\left(t,z^{-1}\right)\hat{H}\left(t,z^{-1}\right)+\hat{B}\left(t,z^{-1}\right)\hat{G}\left(t,z^{-1}\right)$$

where $T\left(z^{-1}\right)=t_{0}+t_{1}z^{-1}+t_{2}z^{-2}$ is a pre-specified characteristic polynomial with stable poles.

Now that the estimates ${\hat{a}}_{1}\left(t\right)$ and ${\hat{b}}_{1}\left(t\right)$ are obtained, then the coefficients $\hat{h}\left(t\right)$ , ${\hat{g}}_{0}\left(t\right)$ , and ${\hat{g}}_{1}\left(t\right)$ can be updated based on the relation Equation (28):


(28) $$\hat{h}\left(t\right)=t_{0},\;{\hat{g}}_{0}\left(t\right)=\frac{t_{1}+t_{0}-{\hat{a}}_{1}\left(t\right)t_{0}}{{\hat{b}}_{1}\left(t\right)},\;{\hat{g}}_{1}\left(t\right)=\frac{t_{2}+{\hat{a}}_{1}\left(t\right)t_{0}}{{\hat{b}}_{1}\left(t\right)}$$

Similar to Equation (10), now the proportional and differential gains in Equation (8) are chosen by the following relation:


(29) $$\left\{ \begin{array}{c} k_{P}+k_{I}=\frac{{\hat{g}}_{0}\left(t\right)}{\hat{h}\left(t\right)}\\ -k_{P}=\frac{{\hat{g}}_{1}\left(t\right)}{\hat{h}\left(t\right)} \end{array}\right.$$

which means that *k_p_* and *k_I_* should be designed as:


(30) $$k_{P}=-\frac{t_{2}+{\hat{a}}_{1}\left(t\right)t_{0}}{{\hat{b}}_{1}\left(t\right)t_{0}},\;k_{I}=\frac{t_{1}+t_{2}+t_{0}}{{\hat{b}}_{1}\left(t\right)t_{0}}$$

From Equation (30), it is found that ${\hat{b}}_{1}\left(t\right)$ appears in the denominator. In order to ensure the smoothness of the control law, we impose a constrain on ${\hat{b}}_{1}\left(t\right)$ :


(31) $${\hat{b}}_{1}\left(t\right)=\{\begin{array}{c} \overset{⌢}{b}\;\;\mathrm{if}\;{\hat{b}}_{1}\left(t\right)\leq \overset{⌢}{b}\\ \begin{array}{cc} {\hat{b}}_{1}\left(t\right) & \mathrm{else} \end{array} \end{array}$$

where $\overset{⌢}{b}$ is a pre-specified upper bound. It is noted that such treatment has no negative effect on the convergence or stability properties (Chen et al., 2001).

The proposed PI controller can be implemented as follows:

*Step 1*: Update ${\hat{a}}_{1}\left(t\right)$ and ${\hat{b}}_{1}\left(t\right)$ by Equations (16)–(21);

*Step 2*: Calculate $\hat{h}\left(t\right)$ , ${\hat{g}}_{0}\left(t\right)$ and ${\hat{g}}_{1}\left(t\right)$ by Equation (28);

*Step 3*: Calculate *k_p_* and *k_I_* by Equation (30);

*Step 4*: Calculate *u*(*t*) by Equation (8);

*Step 5*: Let *t* =*t* + 1 and apply *u*(*t*) to the plant.

### 2.4 Human–robot interaction control framework

**Figure 4:**
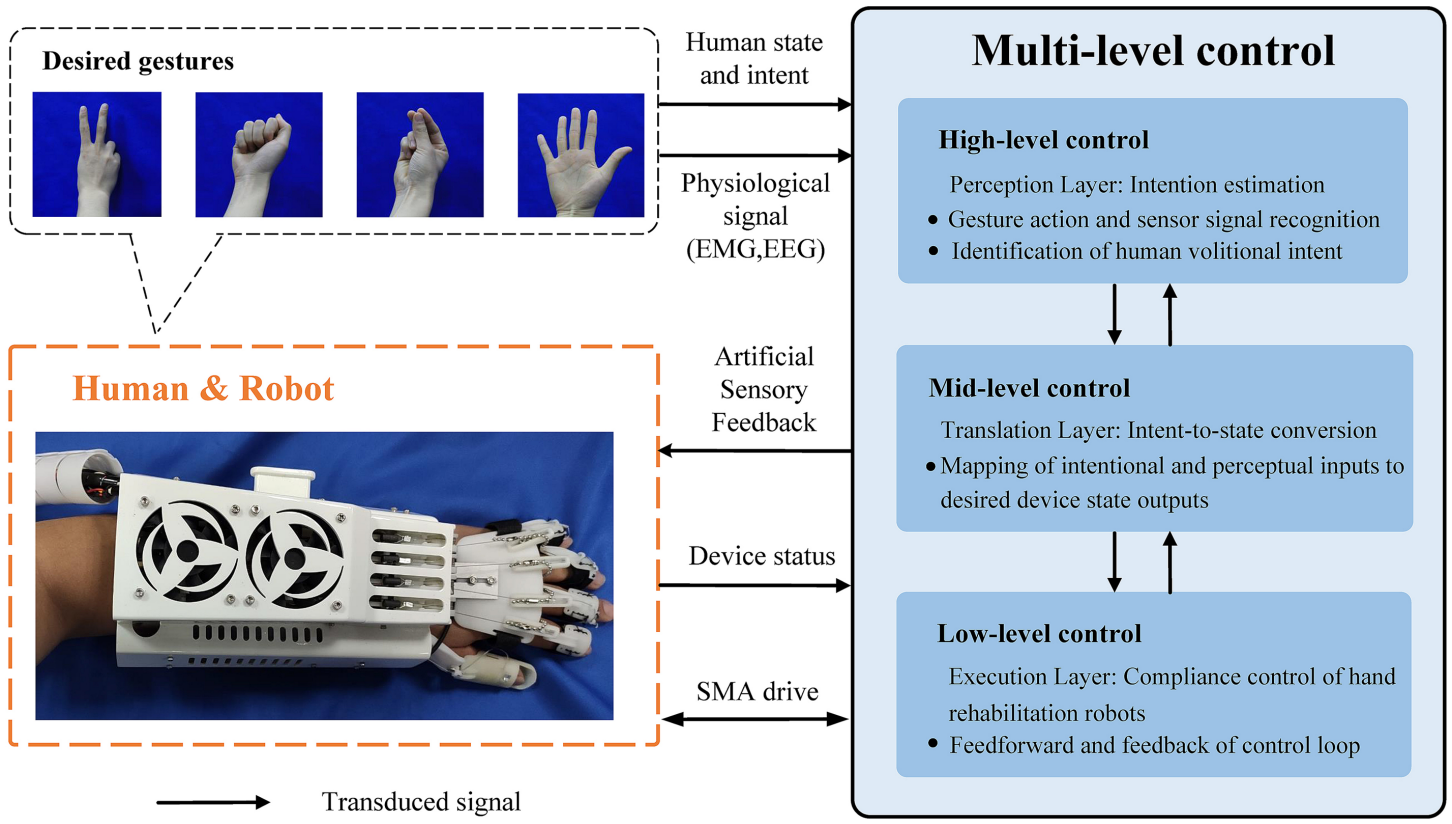
A framework for human–robot-environment interaction control strategy.

The integration of voluntary participation and mechanical assistance in robot-assisted rehabilitation for hand rehabilitation is also crucial. Therefore, a SMA actuator-based rehabilitation robotic system is needed to not only perform motion-guided training for functional rehabilitation of patients with impaired hand function, but also to assist and collaborate with the patient’s preserved motor abilities to achieve on-demand assistance. Therefore, collaboration and interaction between the patient and the rehabilitation robot during human–robot interaction is a major challenge for the control system. To address this challenge, we plan to propose a fusion human–robot-environment interaction control framework that incorporates multi-level control research techniques. The framework for human–robot-environment cohesive interaction control strategy is shown in Figure 4.

The intention for motion is generated by the user themselves, requiring the recognition of human intent. User states include the body’s posture, velocity, and the physical interactions between the user, environment, and devices. The external environment comprises spatial features and terrain, which the controller can also perceive and take into consideration. At the high level, the controller needs to perceive human intent, recognize mental thoughts, and perform pattern recognition for gesture activities such as clenching fists or bending fingers. In the mid-level, the user’s intent is translated into the desired state of the device by adjusting controller gains, switching models, or tuning model parameters. At the low level, the device’s controller and specific control algorithms are responsible for realizing the desired device state and achieving compliant motion control of the hand rehabilitation robot. Finally, the SMA actuators-based hand rehabilitation robot executes control commands to achieve hand rehabilitation for the user. The hand rehabilitation robot system could also provide artificial sensory feedback in combination with pre-set electrical stimulation, etc.

In the research conducted in this paper, our proposed adaptive control method focuses on robust adaptive control at the low level controller of the SMA actuator-based hand rehabilitation robots. This method ensures stability and practicality during human–robot interactions. The experimental verification process will be presented in the following sections. It is worth pointing out that in our proposed control framework for human–robot interaction, the research methods related to mid-level and high-level control are already relatively mature. For instance, our research team has proposed a continuous estimation method for upper limb multi-joint motions based on sEMG (Ding et al., 2016). Moreover, deep learning has recently been widely applied in sEMG signal recognition and gesture classification (Xiong et al., 2021). In addition, some research teams have proposed methods for electrode shifts estimation and adaptive correction, applying them to enhance the robustness of sEMG recognition in hand rehabilitation processes (Li et al., 2020). Besides, a benchmark dataset of sEMG in non-ideal conditions (SeNic) has also been introduced to investigate the robustness of gesture recognition based on surface electromyographic signals in practical applications (Zhu et al., 2022). In summary, extensive research has been conducted on mid-level and high-level control for hand neurorehabilitation. Therefore, due to space constraints, we will not elaborate further on this aspect.

## 3 Experiments and results

### 3.1 Experimental validation on the SMA actuator-based platform

#### 3.1.1 The experimental set-up

**Figure 5:**
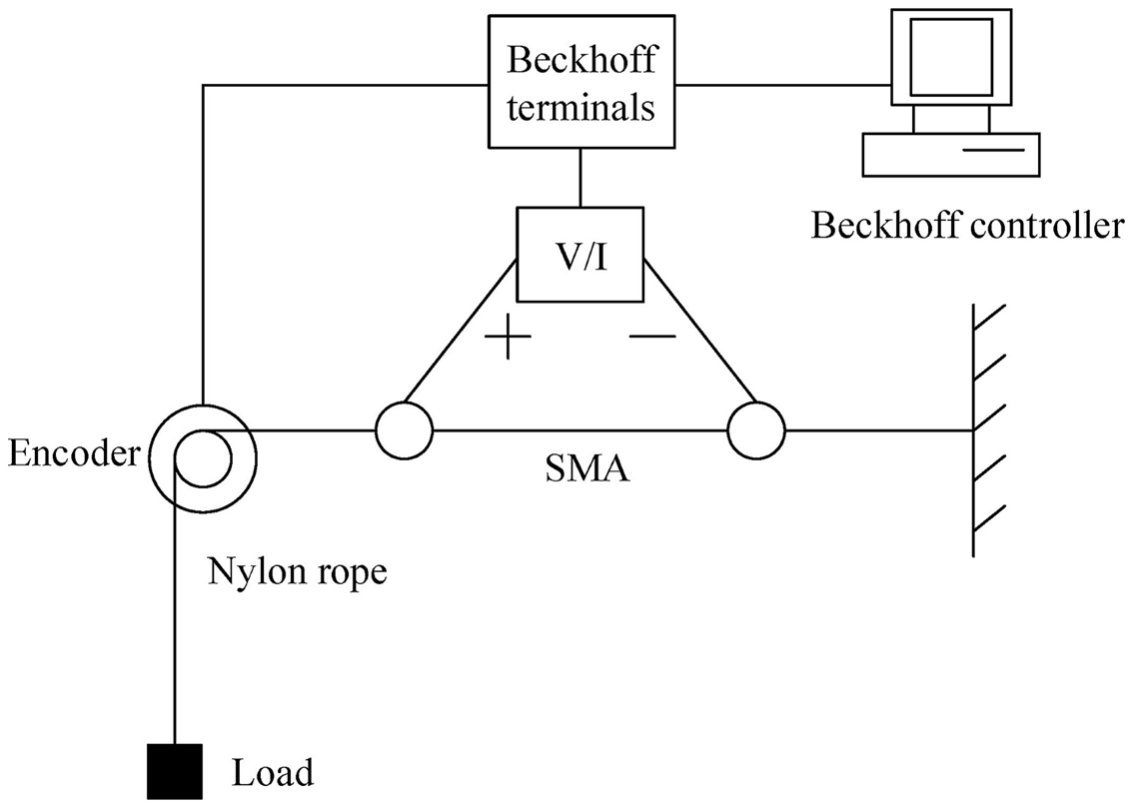
The experimental platform diagram of the SMA actuator.

**Figure 6:**
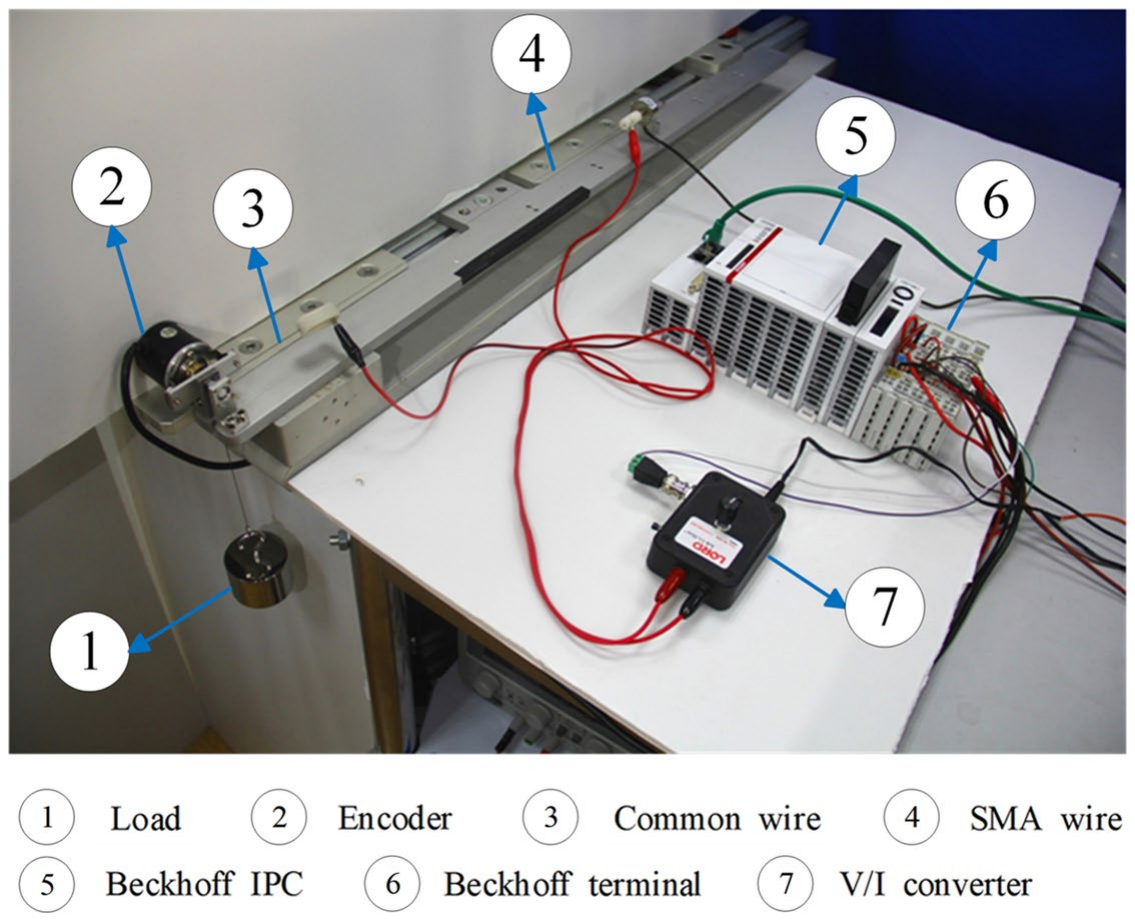
The experimental set-up of the SMA actuated system.

The experimental platform diagram of the SMA actuator is depicted in Figure 5, and the experimental set-up is presented in Figure 6. The structure of this SMA actuated system is similar to the one in Romano and Tannuri (2009) but without a cooling device. The SMA wire is the Flexinol actuator wire which is produced by Dynalloy, Inc. For this type of wire, the diameter is 0.25 mm, the length is 340 mm, the deformations are up to about 4%, and the Austenite start temperature is 90°C. In this experiment, the system output is the displacement (unit: *m*) and the input signal is the current (unit: *A*), which is constrained to the range 0 ~ 0.4. The control current applied to the SMA actuator is obtained from a V/I converter. The SMA wire then generates significant strains in response to the temperature changes caused by the current heating effect. The displacement of the SMA wire is measured by a high precision encoder. The Beckhoff EtherCAT terminals are used for the transformation and conversion of data, and the sample frequency is 200 Hz. The load is fixed as 500 *g* for the set-point tracking experiment, but varies for the other experiments.

To describe this nonlinear plant, two groups of models have been considered in previous studies (Nikdel et al., 2014; Pai et al., 2017; Pan et al., 2017), namely, mechanism models or neural networks models. However, there exist some inevitable drawbacks in each group. The objective of this work is to find an alternative way to simultaneously address the computational burden and the unmodeled dynamics issues.

#### 3.1.2 PI controllers design

The proposed adaptive PI controller is applied to this plant. Before the control implementation, some offline identifications have been carried out in the Matlab software. The purpose of the offline procedure is to probe the main dynamic properties of this nonlinear plant. Based on some groups of input–output data around different operating points, an RLS algorithm is used to identify the parameters of the model Equation (6). Then some groups of convergent parameter estimates are obtained. Based on these estimates and other input–output data, we have also conducted the model test experiment. Finally, the best prediction model is selected as *y*(*t* + 1) = 0.9923 *y*(*t*) + 0.001 *u*(*t*). Meanwhile, the obtained results are used as initial conditions for the controller design. For the proposed PI control method, the initialization is $\hat{\theta }\left(0\right)={\left[-0.9923,0.001\right]}^{T}$ , the multi-innovation length is *p* = 3, the gain is ε = 1, the bound is Δ = 0.00012, the characteristic polynomial is pre-specified as $T\left(z^{-1}\right)=1-1.44z^{-1}+0.445z^{-2}$ , and the constrain is $\overset{⌢}{b}=0.001$ .

As a comparison, the conventional fixed-gains PI controller is applied to this plant as well. The proportional and differential gains *k_p_* and *k_I_* are pre-specified as *k_p_* = 500 and *k_p_* = 5.

#### 3.1.3 Set-point tracking

**Figure 7:**
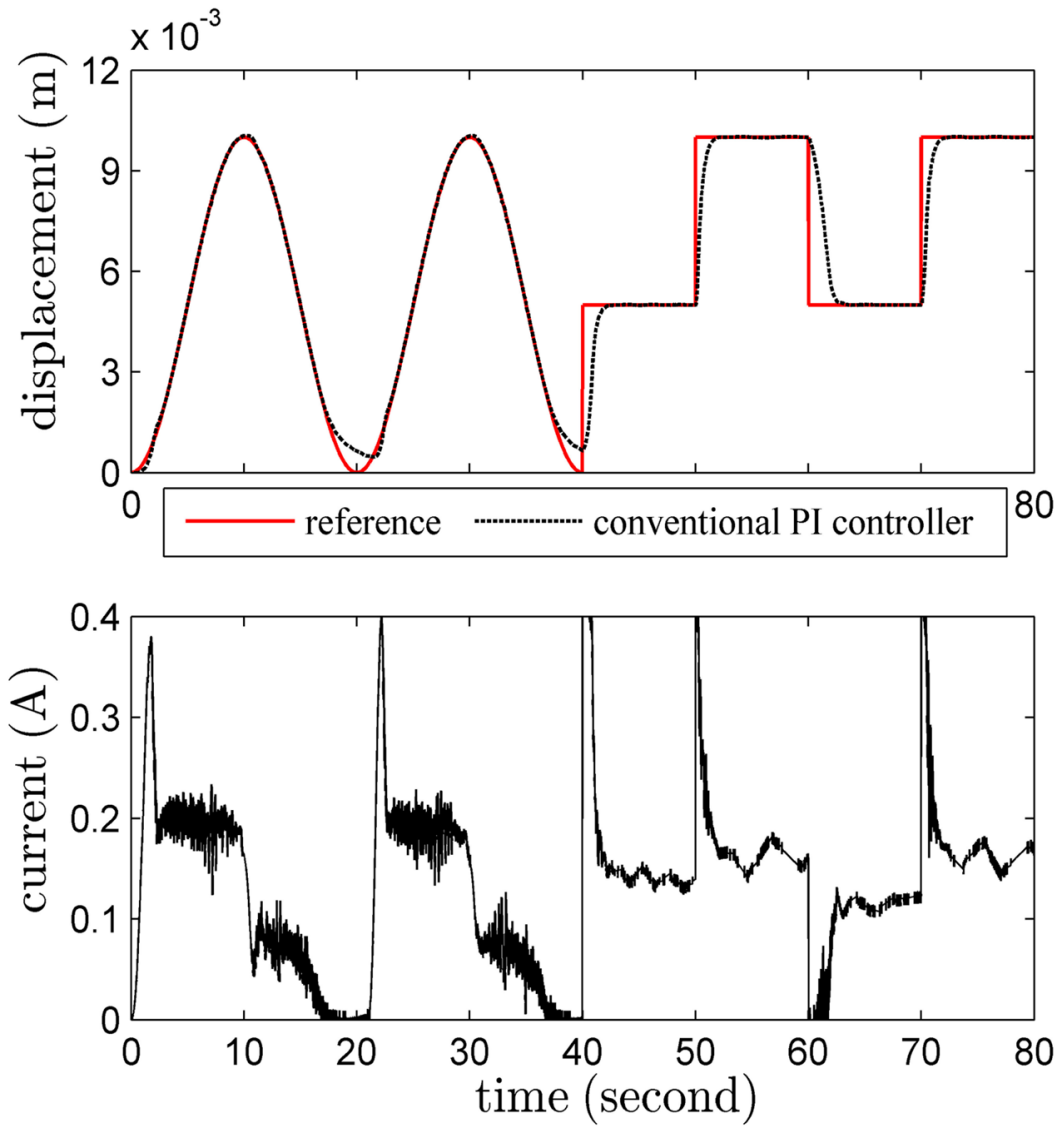
Set-point tracking results by the conventional PI scheme.

The load is fixed as 500 *g* in this test. Sinusoidal trajectory and square-wave trajectory are both considered. The set-point tracking results of these methods are shown in Figures 7 8.

**Figure 8:**
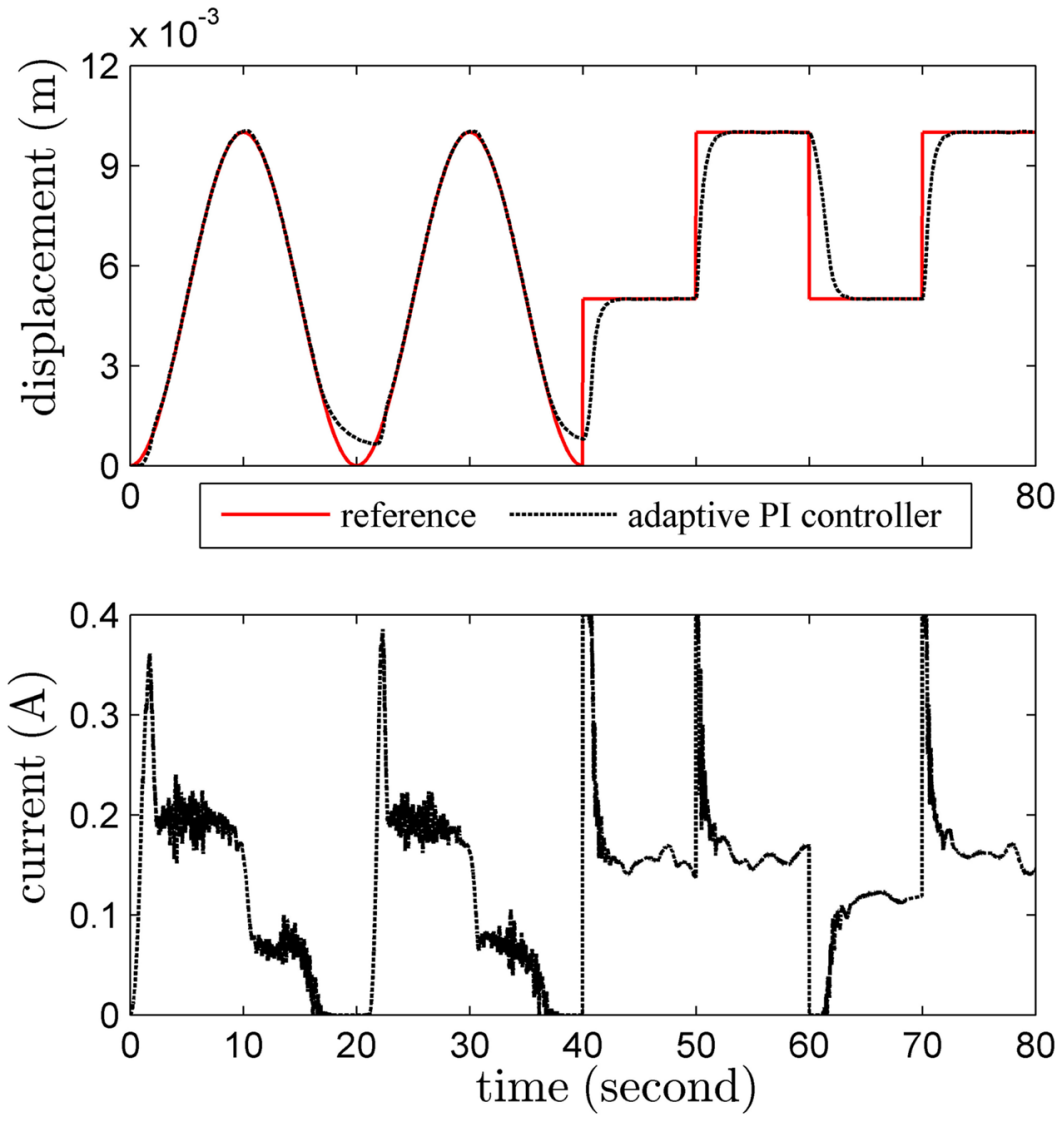
Set-point tracking results by the adaptive PI scheme.

The performance of the adaptive PI controller is better than the conventional PI one, especially for the milder control input. It is obvious that the adaptive PI controller can accurately track the reference trajectory with a slowly changing reference trajectory. In addition, the overshoot and oscillation of the adaptive PI controller are more satisfactory. Interestingly, the unmodeled dynamics has been gradually compensated by the adaptive PI controller, which can verify Theorem 1 in Appendix.

#### 3.1.4 Load variations

**Figure 9:**
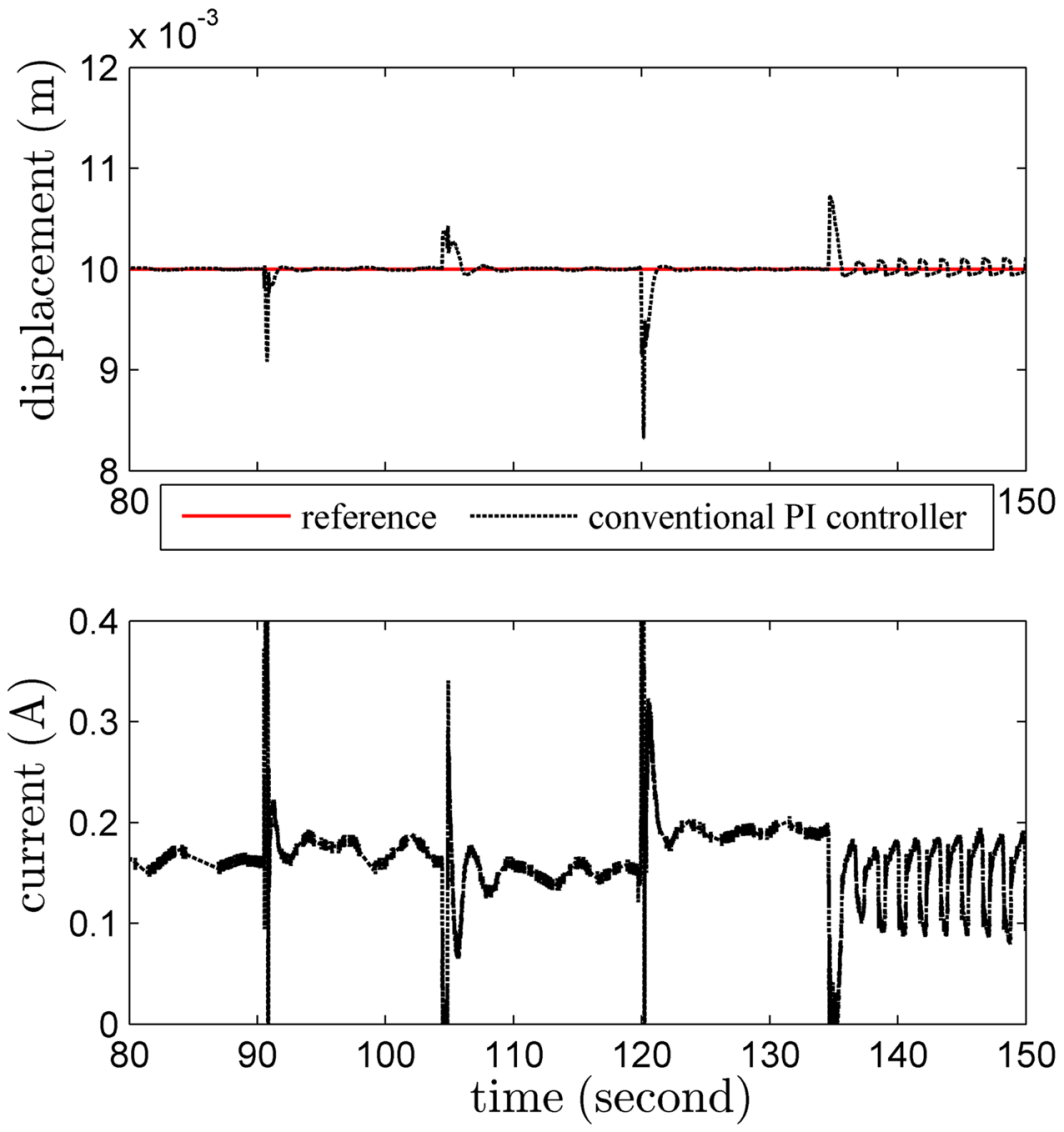
Regulation results under load variations by the conventional PI scheme.

An additional load with 200 *g* is imposed on this actuator at 90th second, and removed at 105th second. Another heavier load with 300 *g* is added at 120th second, and removed at 135th second. The regulation results are shown in Figures 9 10.

**Figure 10:**
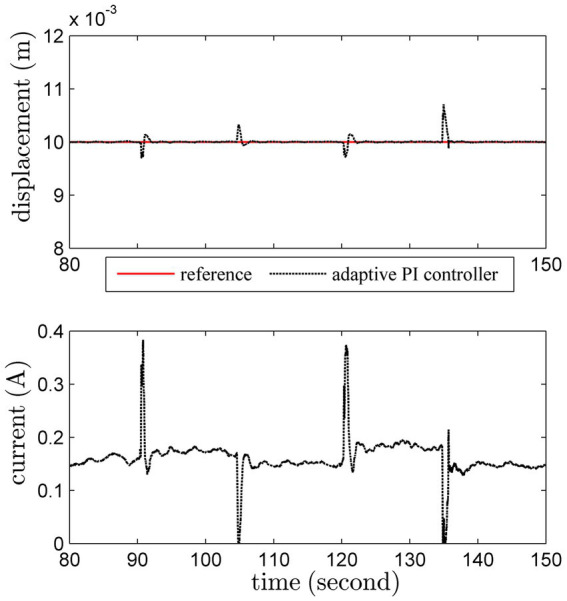
Regulation results under load variations by the adaptive PI scheme.

The conventional PI controller leads to unattractive results under uncertainties induced by load variations. Worse still, the system becomes unstable after 135th second. Obviously, thanks to the online adaptation, the adaptive PI controller can ensure satisfactory robust stability despite of severe uncertainties.

#### 3.1.5 Disturbance rejection

**Figure 11:**
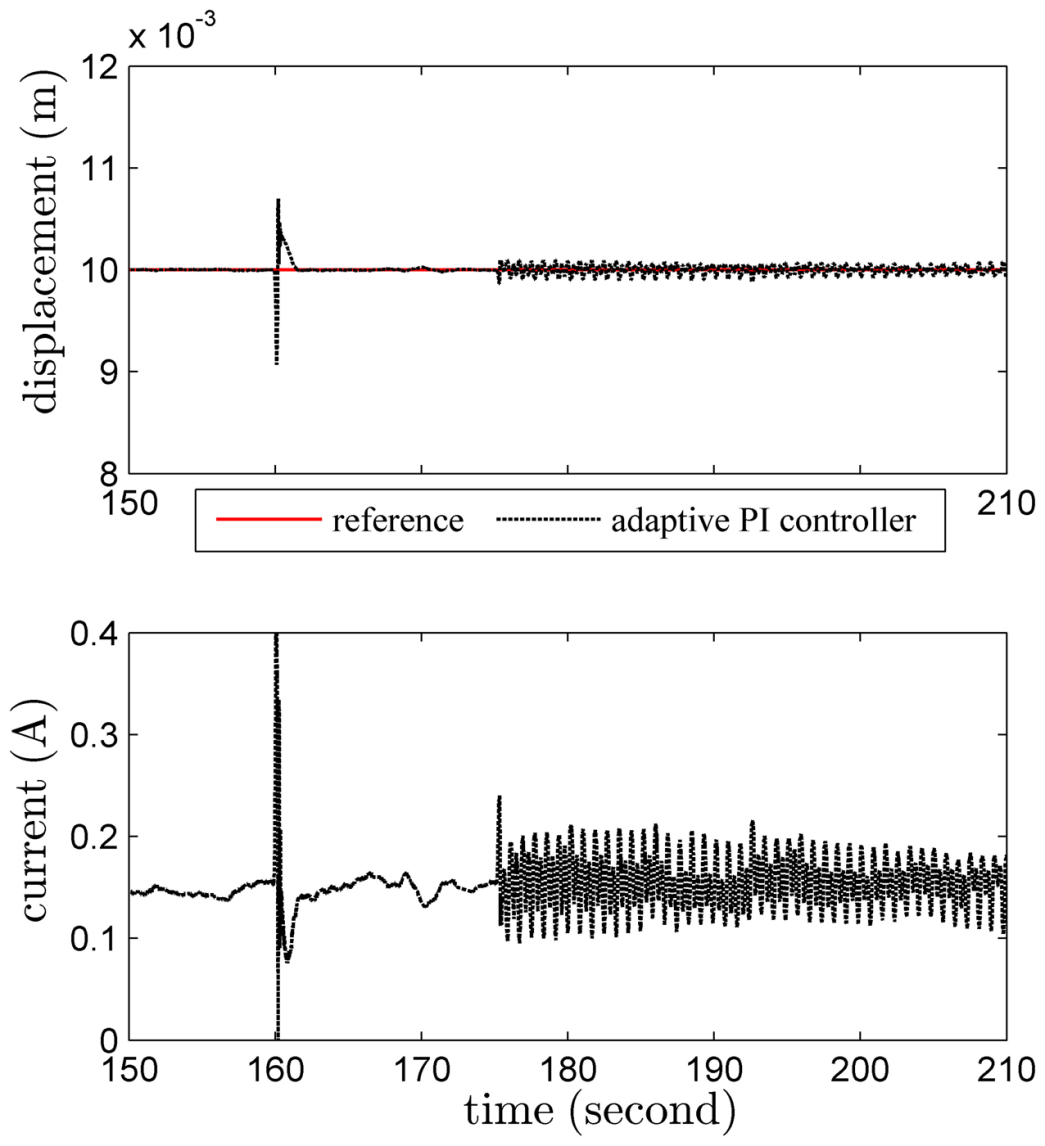
Regulation results under non-Gaussian by the adaptive PI scheme.

We further test the disturbance rejection ability. An unknown instantaneous vertical force is suddenly imposed on the load at 160th second, and then an unknown instantaneous lateral force is suddenly added at 175th second. The disturbance rejection result of the proposed adaptive PI controller is shown in Figure 11.

The result shows that the proposed adaptive PI controller is reliable under non-Gaussian stochastic noise, which is ensured by the dead-zone weighted factor Equation (20). Though the control input varies a lot, the system output stays within a small region.

#### 3.1.6 Summary

**Figure 12:**
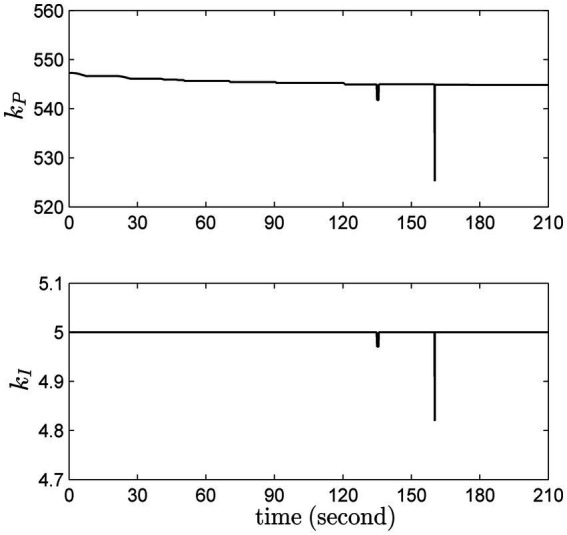
The online updates of the proportional and differential gains.

For robotic applications, plenty of issues (i.e., modeling error, load variations and stochastic noise) may cause uncertainties. The proposed adaptive PI controller can address these issues in a computationally efficient manner. During the whole operation, the proportional and differential gains *k_p_* and *k_I_* are updated according to the current working conditions, as shown in Figure 12. Most interestingly, it is seen that when the system suffers from severe uncertainties, especially around 135th and 160th seconds, the updated gains can address the negative effects timely.

### 3.2 Experiments on SMA actuator-based hand rehabilitation robot system

#### 3.2.1 The experimental set-up

**Figure 13:**
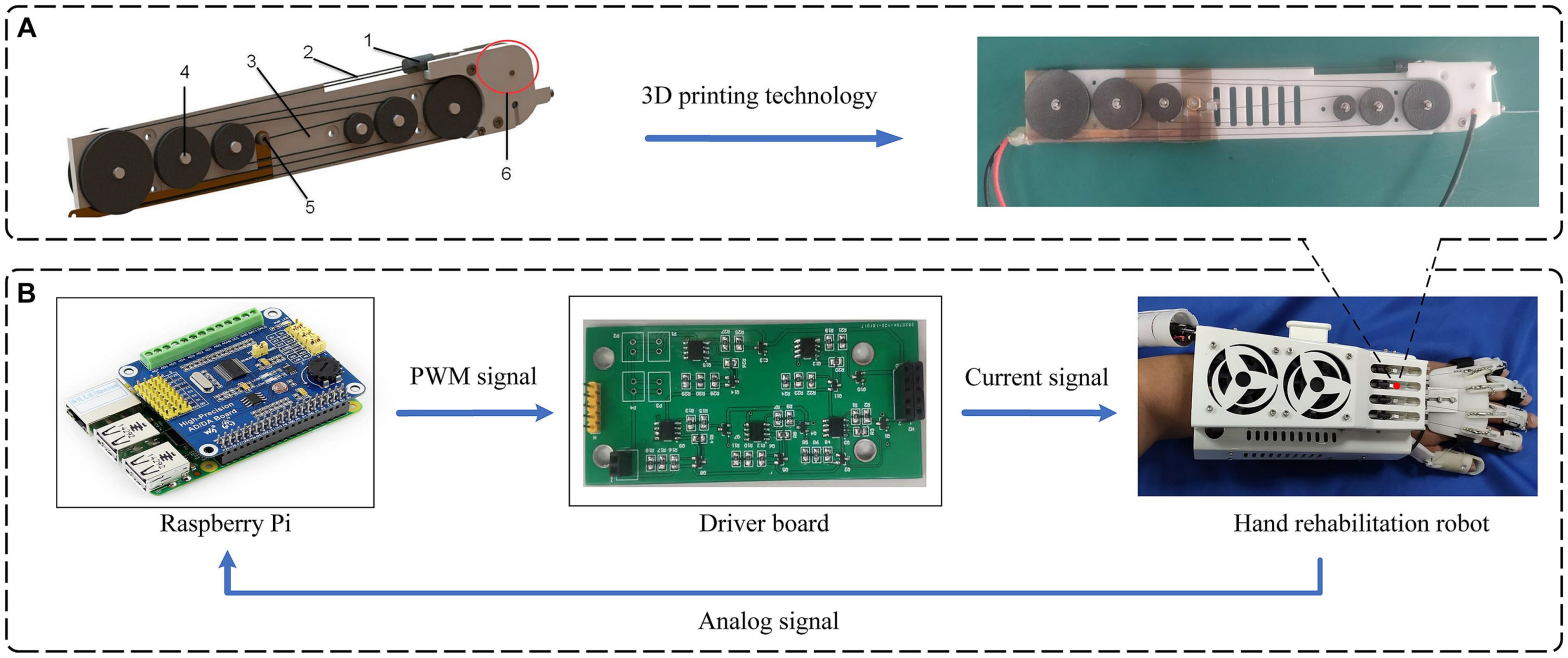
Hand rehabilitation robotic system based on SMA actuator. **(A)** SMA actuator structure diagram: 1- Connection, 2- SMA Wires, 3- Actuator body plate, 4- Pulley mechanism, 5- Wiring mechanism, 6- Displacement feedback mechanism. **(B)** System hardware integration framework for hand rehabilitation robot.

After verifying the driving principle of SMA and the proposed adaptive PI control algorithm, we designed an actuator mechanism based on SMA and integrated it into a hand rehabilitation robot system to form an SMA-based hand rehabilitation robot system platform, which is suitable for hand rehabilitation training of hemiplegic patients. In this hand rehabilitation robot, each finger is controlled by an individual SMA actuator, and the entire robot comprises five identical SMA actuators. The SMA actuator is primarily comprised of six components, as shown in the left part of Figure 13A. This includes the installation of a pulley device on the main plate of the actuator, winding a shape memory alloy wire around the pulley, connecting the shape memory alloy wire to the output wire and the preloaded pulley through connecting members, and incorporating a wiring mechanism on the actuator’s main plate for ease of wiring. Additionally, a displacement feedback mechanism is established to enhance control over the shape memory alloy wire. A prototype SMA actuator was fabricated and assembled using 3D printing technology, as shown in the right part of Figure 13A. To prevent short-circuiting of the wiring mechanism with the shape memory alloy filament, a layer of Teflon tape with insulating and high-temperature-resistant properties was applied to the copper sheet of the wiring mechanism.

After the hand rehabilitation robot system based on SMA actuator is built, the movement of the hand rehabilitation robot is controlled in the form of sending commands from the upper computer to the lower computer, so as to assist the patient in rehabilitation training. The framework of the hardware system is shown in Figure 13B.

#### 3.2.2 Position tracking experiments of hand rehabilitation robot system based on adaptive PI control

**Figure 14:**
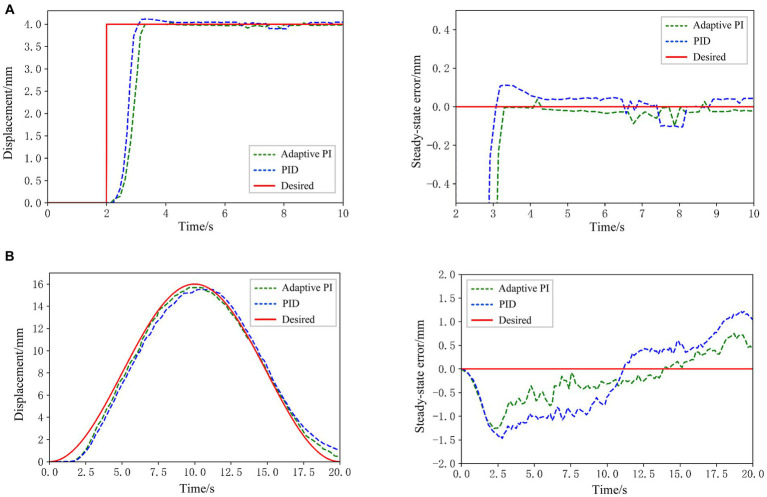
**(A)** Position tracking result and system steady state error for step signal tracking. **(B)** Position tracking result and system steady state error for and sinusoidal signal tracking.

In this subsection, the control core utilizes the Raspberry Pi, and the SMA is subjected to heating signals dispatched to the controller, causing it to contract and deform, thereby propelling the movement of the rehabilitation hand. In this experiment, the SMA actuator of the index finger part of the hand rehabilitation robot is selected as the control object, and based on the adaptive PI control algorithm proposed in this paper to track the position response curve of the SMA actuator under the step signal as well as the sinusoidal signal. For comparative analysis, the PID control law (Khalil, 1996) is utilized as a reference algorithm. Meanwhile, in order to be able to visually compare and analyze the control effects of the two control algorithms, the errors of the SMA actuator-based hand rehabilitation robotic system will be compared when it reaches the steady state under the two control algorithms, respectively. The actual results of the robot system tracking the step and sinusoidal signals and the steady state error results are shown in Figure 14.

From the experimental results in Figure 14A, it can be seen that under the adaptive PI control algorithm, the desired value of the hand rehabilitation robot system is set to 4 mm at 2 s, and the system responds at 2.4 s, reaches the desired position at about 3 s, and maintains stability thereafter, with almost no deviation. Meanwhile, the response times of the two control algorithms are basically the same, but the hand rehabilitation robotic system does not produce overshooting and has a smaller steady state error when the step signal is tracked under the adaptive PI control algorithm. Consequently, for reference trajectories represented by step signals, the hand rehabilitation robot system demonstrates superior control performance under the adaptive PI control algorithm proposed in this paper. Examining the experimental outcomes in Figure 14B, it is observed that the hand rehabilitation robotic system adeptly tracks sinusoidal signals. While the response times of the SMA actuator system remain consistent under both algorithms, the adaptive PI algorithm proposed in this paper achieves more accurate position tracking with less error when tracking sinusoidal signals. Thus, for various signal amplitudes, the methodology presented in this paper enables the SMA actuator-based hand rehabilitation robotic system to approach the target position with reduced overshooting and a smaller steady-state error. These experiments substantiate the reliability and accuracy of the proposed methodology, affirming the safety of the SMA actuator-based hand rehabilitation robot in assisting subjects during the rehabilitation training process.

#### 3.2.3 Experiments on hand rehabilitation training with different gestures

**Figure 15:**
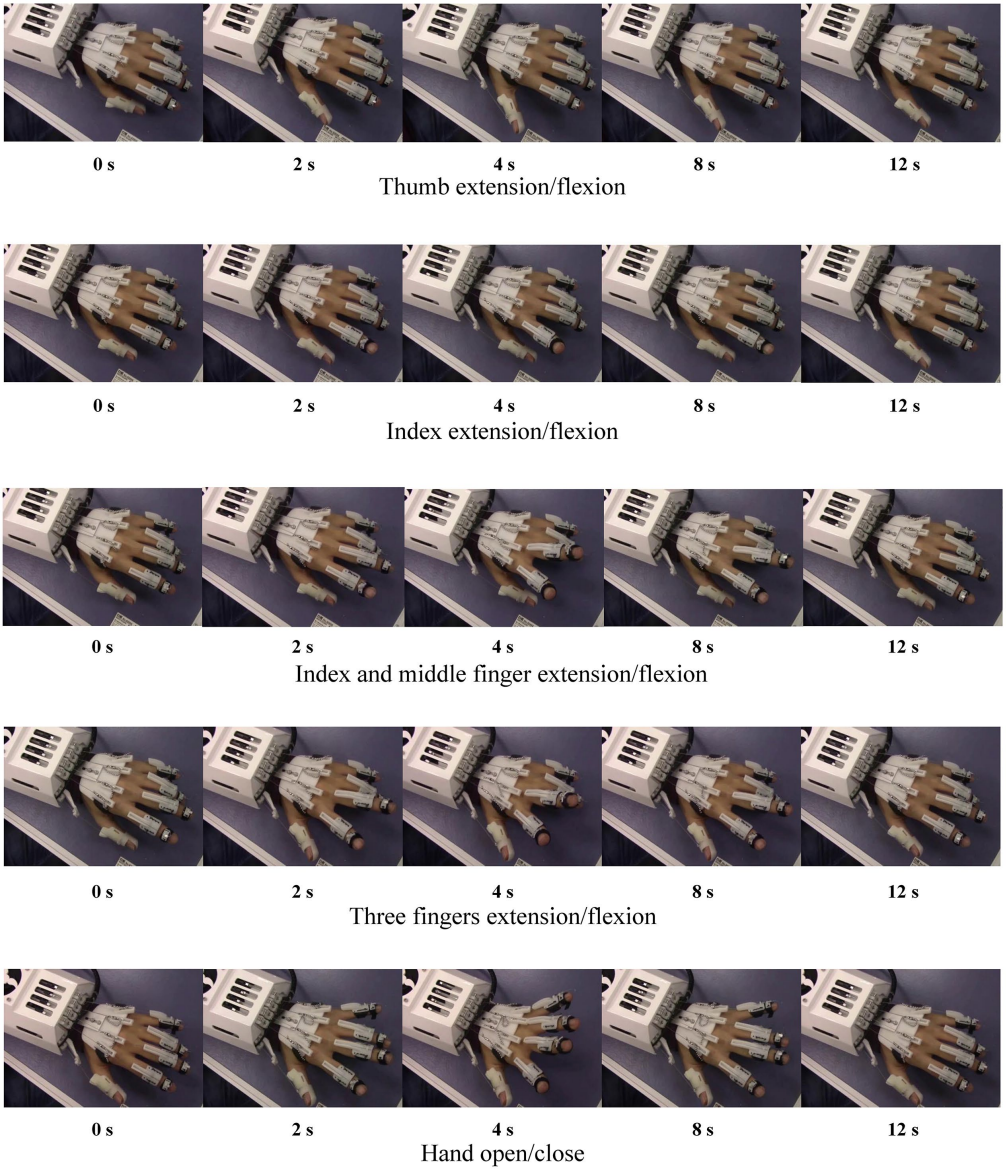
Passive rehabilitation of the hand with different gestures.

We oriented the SMA actuator-based hand rehabilitation robotic system platform to conduct the hand passive rehabilitation training experiments on subjects with different gestures, and the training process is shown in Figure 15. The hand rehabilitation exercises are divided into five movements, which are thumb extension/flexion, index extension/flexion, index and middle finger extension/flexion, three fingers extension/flexion and hand open/close. During the hand gesture rehabilitation training experiment with the SMA actuator-based hand rehabilitation robot system, a complete single flexion-extension training cycle takes a total of 12 s. Throughout this process, spanning from 0 to 4 s, the SMA contracts upon heating and powering, propelling the fingers to their maximum extended position. Subsequently, from 4 to 12 s, the SMA undergoes cooling facilitated by a fan on the outer shell of the hand rehabilitation robot, causing the hand to return to its initial state. Importantly, this mechanism satisfies the requirements of passive rehabilitation training for multiple gestures in patients with hand hemiplegia, demonstrating an optimal control effect. This experiment effectively establishes the reliability and precision of the SMA actuator-based hand rehabilitation robotic system for subject-specific rehabilitation training under the adaptive PI control strategy. It is worth noting that, due to space limitations, our experiments only focused on the low-level robust adaptive control of hand rehabilitation robots based on SMA actuators. We did not conduct experiments related to neural rehabilitation control involving mid-level and high-level controllers. This aspect will be addressed in our future research.

## 4 Discussion and conclusion

This paper presents an innovative adaptive PI control strategy tailored for SMA actuators. Utilizing a simplified gray-box model, the primary dynamic properties of the plant are approximated. An efficient adaptive algorithm is then introduced to iteratively update the model parameters. Subsequently, a PI control law is proposed, with gains calculated through the pole assignment technique, ensuring closed-loop stability under mild conditions. Notably, the strategy exhibits robustness, particularly in the face of load variations and continuous disturbances. The proposed adaptive control algorithm is well-defined and has been initially experimentally validated on a Beckhoff controller. Finally, the SMA actuator is designed, fabricated and integrated into a hand rehabilitation robot system, and the position tracking experiments of the SMA actuator based on the proposed adaptive PI control strategy are conducted to verify the stability and accuracy of the proposed control algorithm. Meanwhile, rehabilitation training for several different gestures was conducted for subjects to verify the reliability of the hand rehabilitation robot system based on the SMA actuator.

From another perspective, the control method proposed in this paper exhibits closed-loop stability. Additionally, it is based on several foundational assumptions and theorems, as mentioned in Equation (15) and Theorem 1. The assumption in Equation (15) implies treating unmodeled dynamics as bounded disturbances, and parameter estimation can reject certain continuous disturbances. From the practical application standpoint in the field of hand rehabilitation robotics, disturbances within bounds refer to slow temperature changes in the rehabilitation environment or subtle vibrations in the load. Disturbances beyond bounds refer to severe shaking of the load or significant parameter drift. Furthermore, regarding Equation (A20) in Theorem 1, in actual rehabilitation scenarios, especially in hand rehabilitation, the rehabilitation goals and environment are relatively stable systems not subject to large-scale fluctuations. Therefore, Equation (A20) is satisfied according to the practical needs of rehabilitation. For Equation (A21), in practical applications, for the safety of patients, the reference trajectory of rehabilitation equipment changes slowly and has a small range during the hand rehabilitation process. Therefore, we believe that (Equation A21) can be satisfied in practical applications. In summary, from the perspective of practical applications in the rehabilitation field, our system complies with Theorem 1, demonstrating rationality and reliability.

For control issues of SMA actuators, the systematic method derived in this work probably is the simplest adaptive controller so far, which takes into account the model computational complexity as well as the accuracy of the control results, and the controller has good practicability and reliability. In the future, we expect that the theoretical achievements we have obtained can be further applied to a broader range of rehabilitation robotic devices.

## Data availability statement

The raw data supporting the conclusions of this article will be made available by the authors, without undue reservation.

## Ethics statement

The studies involving humans were approved by the Research Ethics Committee of the Shenyang Institute of Automation. The studies were conducted in accordance with the local legislation and institutional requirements. The participants provided their written informed consent to participate in this study. The animal study was approved by the Research Ethics Committee of the Shenyang Institute of Automation. The study was conducted in accordance with the local legislation and institutional requirements. Written informed consent was obtained from the individual(s) for the publication of any potentially identifiable images or data included in this article.

## Author contributions

ES: Conceptualization, Formal analysis, Methodology, Validation, Writing – original draft. XZ: Data curation, Investigation, Methodology, Writing – original draft. TW: Project administration, Resources, Visualization, Writing – review & editing. XL: Funding acquisition, Project administration, Supervision, Writing – review & editing. CB: Resources, Software, Supervision, Writing – review & editing. XZ: Conceptualization, Funding acquisition, Project administration, Resources, Writing – review & editing.

## References

[ref1] Airoldi G. Riva G. Ranucci T. Vicentini B. (1991). Electric transport properties of a NiTi shape memory alloy under applied stress. MRS Online Proc. Libr. Arch. 246, 277–281. doi: 10.1557/PROC-246-277

[ref2] Ashrafiuon H. Jala V. R. (2009). Sliding mode control of mechanical systems actuated by shape memory alloy. ASME J. Dyn. Syst. Meas. Control. 131, 101–116. doi: 10.1115/1.3023121

[ref3] Chen X. K. (2006). Adaptive sliding mode control for discrete-time multi-input multi-output systems. Automatica 42, 427–435. doi: 10.1016/j.automatica.2005.10.008

[ref4] Chen X. K. Fukuda T. Young K. D. (2001). Adaptive quasi-sliding-mode tracking control for discrete uncertain input-output systems. IEEE Trans. Ind. Electron. 48, 216–224. doi: 10.1109/41.904582

[ref5] Deberg L. Taheri Andani M. Hosseinipour M. Elahinia M. (2014). An SMA passive ankle foot orthosis: design, modeling, and experimental evaluation. Smart Mater. Res. 11:572094. doi: 10.1155/2014/572094

[ref6] Ding F. Chen T. W. (2006). Performance analysis of multi-innovation gradient type identification methods. Automatica 43, 1–14. doi: 10.1016/j.automatica.2006.07.024

[ref7] Ding Q. Han J. Zhao X. (2016). Continuous estimation of human multi-joint angles from sEMG using a state-space model. IEEE Trans. Neural Syst. Rehabil. Eng. 25, 1518–1528. doi: 10.1109/TNSRE.2016.2639527, PMID: 28113324

[ref8] Elahinia M. H. Ashrafiuon H. (2002). Nonlinear control of a shape memory alloy actuated maniputor. ASME J. Dyn. Syst. Meas. Control. 124, 566–575. doi: 10.1115/1.1501285

[ref9] Fu Y. Chai T. Y. (2011). Indirect self-tuning control using multiple models for non-affine nonlinear systems. Int. J. Control. 84, 1031–1040. doi: 10.1080/00207179.2011.588960

[ref10] Goodwin G. C. Sin K. S. (1984). Adaptive filtering, prediction and control. Englewood Cliffs, New Jersey: Prentice Hall.

[ref11] Jeong J. Hyeon K. Han J. Park C. H. Ahn S. Y. Bok S. K.. (2022). Wrist assisting soft wearable robot with stretchable coolant vessel integrated SMA muscle. IEEE/ASME Trans. Mechatr. 27, 1046–1058. doi: 10.1109/TMECH.2021.3078472

[ref12] Jeong J. Yasir I. B. Han J. Park C. H. Bok S. K. Kyung K. U. (2019). Design of Shape Memory Alloy-Based Soft Wearable Robot for assisting wrist motion. Appl. Sci. 9:4025. doi: 10.3390/app9194025

[ref13] Khalil H. K. (1996). Adaptive output feedback control of nonlinear systems represented by input-output models. IEEE Trans. Autom. Control 41, 177–188. doi: 10.1109/9.481517

[ref14] Kumbhar S. B. Chavan S. P. Gawade S. S. (2017). Adaptive tuned vibration absorber based on magnetorheological elastomer-shape memory alloy composite. Mech. Syst. Signal Process. 100, 208–223. doi: 10.1016/j.ymssp.2017.07.027

[ref15] Lagoudas., Dimitris C., ed. (2008). Shape memory alloys: modeling and engineering applications. Berlin: Springer Science & Business Media.

[ref16] Lai J. Song A. Shi K. Ji Q. Lu Y. Li H. (2023). Design and evaluation of a bidirectional soft glove for hand rehabilitation-assistance tasks. IEEE Trans. Med. Robot. Bion. 5, 730–740. doi: 10.1109/TMRB.2023.3292414

[ref17] Li Z. Zhao X. Liu G. Zhang B. Zhang D. Han J. (2020). Electrode shifts estimation and adaptive correction for improving robustness of sEMG-based recognition. IEEE J. Biomed. Health Inform. 25, 1101–1110. doi: 10.1109/JBHI.2020.3012698 32750979

[ref18] Mataee M. G. Taheri Andani M. Elahinia M. (2015). Adaptive ankle–foot orthoses based on superelasticity of shape memory alloys. J. Intell. Mater. Syst. Struct. 26, 639–651. doi: 10.1177/1045389X14544145

[ref19] Nikdel N. Nikdel P. Badamchizadeh M. A. Hassanzadeh I. (2014). Using neural network model predictive control for controlling shape memory alloy-based manipulators. IEEE Trans. Ind. Electron. 61, 1394–1401. doi: 10.1109/TIE.2013.2258292

[ref20] Pai A. Riepold M. Trächtler A. (2017). Model-based precision control and force control of SMA actuators with a clamping application. Mechatronics 50, 303–320. doi: 10.1016/j.mechatronics.2017.08.011

[ref21] Pan Y. P. Guo Z. Li X. Yu H. Y. (2017). Output-feedback adaptive neural control of a compliant differential SMA actuator. IEEE Trans. Control Syst. Technol. 25, 2202–2210. doi: 10.1109/TCST.2016.2638958

[ref22] Pittaccio S. Garavaglia L. Ceriotti C. Passaretti F. (2015). Applications of shape memory alloys for neurology and neuromuscular rehabilitation. J. Funct. Biomater. 6, 328–344. doi: 10.3390/jfb6020328, PMID: 26023790 PMC4493515

[ref23] Riccardi L. Naso D. Turchiano B. Janocha H. (2013). Adaptive control of positioning systems with hysteresis based on magnetic shape memory alloys. IEEE Trans. Control Syst. Technol. 21, 2011–2023. doi: 10.1109/TCST.2012.2222645

[ref24] Romano R. Tannuri E. A. (2009). Modeling, control and experimental validation of a novel actuator based on shape memory alloys. Mechatronics 19, 1169–1177. doi: 10.1016/j.mechatronics.2009.03.007

[ref25] Serrano D. Copaci D. Arias J. Moreno L. E. Blanco D. (2023). SMA-based soft Exo-glove. IEEE Robot. Automat. Lett. 8, 5448–5455. doi: 10.1109/LRA.2023.3295994

[ref26] Serrano D. Copaci D. S. Moreno L. Blanco D. (2018). SMA based wrist exoskeleton for rehabilitation therapy. In 2018 IEEE/RSJ international conference on intelligent robots and systems (IROS). 2018 IEEE/RSJ international conference on intelligent robots and systems (IROS) (Madrid, IEEE), 2318–2323.

[ref27] Shariat B. S. Meng Q. Mahmud A. S. Wu Z. Bakhtiari R. Zhang J.. (2017). Functionally graded shape memory alloys: design, fabrication and experimental evaluation. Mater. Des. 124, 225–237. doi: 10.1016/j.matdes.2017.03.069

[ref28] Shi Z. Y. Tian J. W. Luo R. D. Zhao G. Wang T. M. (2017). Multifeedback control of a shape memory alloy actuator and a trial application. IEEE Trans. Syst. Man Cybern. Syst. 48, 1106–1119. doi: 10.1109/TSMC.2016.2641465

[ref29] Son N. N. Anh H. P. H. (2015). Adaptive displacement online control of shape memory alloys actuator based on neural networks and hybrid differential evolution algorithm. Neurocomputing 166, 464–474. doi: 10.1016/j.neucom.2015.03.032

[ref30] Tai N. T. Ahn K. Y. (2010). A RBF neural network sliding mode controller for SMA actuators. Int. J. Control. Autom. Syst. 8, 1296–1305. doi: 10.1007/s12555-010-0615-8

[ref31] Tai N. T. Ahn K. Y. (2012). Output feedback direct adaptive controller for a SMA actuator with a Kalman filter. IEEE Trans. Control Syst. Technol. 20, 1081–1091. doi: 10.1109/TCST.2011.2158435

[ref32] Viscuso S. Pittaccio S. Caimmi M. Gasperini G. Pirovano S. Villa E.. (2009). Pseudoelastic nitinol-based device for relaxation of spastic elbow in stroke patients. J. Mater. Eng. Perform. 18, 805–813. doi: 10.1007/s11665-009-9418-6

[ref33] Wang Y. Zheng S. Pang J. Li S. Li J. (2021). Design and experiment of a hand movement device driven by shape memory alloy wires. J. Robot. 2021, 1–13. doi: 10.1155/2021/6611581

[ref34] Wiest J. H. Buckner G. D. (2014). Indirect intelligent sliding mode control of antagonistci shape memory alloy actuators using hysteretic recurrent neural networks. IEEE Trans. Control Syst. Technol. 22, 921–929. doi: 10.1109/TCST.2013.2272420

[ref35] Wu S. K. Lin H. C. Yen Y. C. (1996). A study on the wire drawing of TiNi shape memory alloys. Mater. Sci. Eng. A. 215, 113–119. doi: 10.1016/09021-5093(96)10369-5

[ref36] Xie Q. Meng Q. Yu W. Wu Z. Xu R. Zeng Q.. (2023). Design of a SMA-based soft composite structure for wearable rehabilitation gloves. Front. Neurorobot. 17:1047493. doi: 10.3389/fnbot.2023.1047493, PMID: 36845070 PMC9950102

[ref37] Xiong D. Zhang D. Zhao X. Zhao Y. (2021). Deep learning for EMG-based human-machine interaction: a review. IEEE/CAA J. Automat. Sin. 8, 512–533. doi: 10.1109/JAS.2021.1003865

[ref38] Zakerzadeh M. R. Sayyaadi H. (2013). Precise position control of shape memory alloy actuator using inverse hysteresis model and model reference adpative control system. Mechatronics 23, 1150–1162. doi: 10.1016/j.mechatronics.2013.10.001

[ref39] Zhang J. Ding F. Shi Y. (2008). Self-tuning control based on multi- innovation stochastic gradient parameter estimation. Syst. Control Lett. 58, 69–75. doi: 10.1016/j.sysconle.2008.08.005

[ref40] Zhu B. Zhang D. Chu Y. Gu Y. Zhao X. (2022). SeNic: an open source dataset for sEMG-based gesture recognition in non-ideal conditions. IEEE Trans. Neural Syst. Rehabil. Eng. 30, 1252–1260. doi: 10.1109/TNSRE.2022.3173708, PMID: 35533170

