# Supplementary material for: Adaptive control for shape memory alloy actuated systems with applications to human–robot interaction
Source: Front Neurosci. 2024 Jan 31;18:1337580. doi: 10.3389/fnins.2024.1337580 (PMC10864648; doi:10.3389/fnins.2024.1337580)
Supplement: Supplementary file 1 [file Data_Sheet_1.pdf]

Appendix

This Appendix analyzes the stability of the closed-loop system.

Definition 1: For the given time-varying polynomials

$$L(t,z^{-1}) = l_0(t) + l_1(t)z^{-1} + \ldots + l_{n_l}(t)z^{-n_l} ,$$

$$M(t,z^{-1}) = m_0(t) + m_1(t)z^{-1} + \ldots + m_{n_m}(t)z^{-n_m} ,$$

we define (Fu and Chai, 2011)

$$L(t,z^{-1})M(t,z^{-1}) := \sum_{i=0}^{n_l} \sum_{j=0}^{n_m} l_i(t)m_j(t)z^{-i-j},$$

$$L(t,z^{-1}) \bullet M(t,z^{-1}) := \sum_{i=0}^{n_l} \sum_{j=0}^{n_m} l_i(t)m_j(t-i)z^{-i-j} .$$

Lemma 1: The adaptation algorithm (16)-(21) ensures the following properties:

$$\lim_{t\to\infty} \frac{\lambda^2(k)\|E(k)\|_2^2}{r(k)} = 0 \quad \text{(A1)}$$

$$\lim_{t\to\infty} ||\hat{\theta}(t) - \hat{\theta}(t-1)|| = 0 \quad \text{(A2)}$$

$\hat{\theta}(t)$ is bounded for any t. (A3)

Proof: Define $V(t) = \tilde{\theta}^T(t)\tilde{\theta}(t)$, where $\tilde{\theta}(t) = \hat{\theta}(t) - \theta$.

Then consider the equation (A4)

$$V(t) - V(t-1) = \frac{2\varepsilon\lambda(t)E^T(t)\Phi^T(t)\tilde{\theta}(t-1)}{r(t)} + \frac{\varepsilon^2\lambda^2(t)E^T(t)\Phi^T(t)\Phi(t)E^T(t)}{r^2(t)} \quad \text{(A4)}$$

From (20), there exists $V(t) = V(t-1)$ when $\|E(t)\|_2 \le \sqrt{p}\Delta$. Now let us consider the case when $\|E(t)\|_2 > \sqrt{p}\Delta$.

For simplicity of the proof, we define the extended output $Y(t) = [y(t), y(t-1), \ldots, y(t-p+1)]^T$, the extended unmodeled dynamics $Z(t) = [\zeta(t), \zeta(t-1), \ldots, \zeta(t-p+1)]^T$. It is clear that $Y(t) = \Phi^T(t)\theta + Z(t)$ and $E(t) = Y(t) - \Phi^T(t)\hat{\theta}(t-1)$, which indicates that $\Phi^T(t)\tilde{\theta}(t-1) = Z(t) - E(t)$. From the definition of $r(t)$ in (19), it is clear that $r(t) = 1 + \sum_{k=1}^{t}\|\Phi(t)\|_2^2 > \|\Phi(t)\|_2^2$.

Then the equation (A4) is written as

$$V(t) - V(t-1) \le \frac{2\varepsilon\lambda(t)E^T(t)(Z(t)-E(t))}{r(t)}$$

$$+\frac{\varepsilon^2\lambda^2(t)\|E(t)\|_2^2\|\Phi(t)\|_2^2}{r^2(t)}$$

$$<\frac{2\varepsilon\lambda(t)\|E(t)\|_2(\|Z(t)\|_2-\|E(t)\|_2)}{r(t)}$$

$$+\frac{\varepsilon^2\lambda^2(t)\|E(t)\|_2^2}{r(t)} \quad \text{(A5)}$$

According to *Assumption 1*, it is found that $\|Z(t)\|_2 \leq \sqrt{p}\Delta$. From (20), it is also known that $\sqrt{p}\Delta - \|E(t)\|_2 = -\lambda(t)\|E(t)\|_2$.

Based on these facts, we have

$$V(t)-V(t-1)<\frac{-2\varepsilon\lambda^2(t)\|E(t)\|_2^2}{r(t)}+\frac{\varepsilon^2\lambda^2(t)\|E(t)\|_2^2}{r(t)}$$

$$=-\frac{(2-\varepsilon)\varepsilon\lambda^2(t)\|E(t)\|_2^2}{r(t)} \quad \text{(A6)}$$

Therefore in order to guarantee the existence of the relation $V(t)-V(t-1)<0$, the coefficient ε must satisfy $0<\varepsilon<2$.

Then there exists that

$$\sum_{k=1}^{t}(V(k)-V(k-1)) \leq -(2-\varepsilon)\varepsilon\sum_{k=1}^{t}\frac{\lambda^2(k)\|E(k)\|_2^2}{r(k)} \quad \text{(A7)}$$

Now combining (A7) with the fact $V(t)\geq 0$ and following the same line as that in Goodwin and Sin (2008), the conclusions (A1)-(A3) can be proven.

Lemma 2: Let *Assumption* 1 hold and apply the proposed controller to the nonlinear system (1), then there exist positive constants K_1, K_2 such that

$$\max_{0\leq\tau\leq t}\|\Phi(\tau)\|_2 \leq K_1 + K_2\max_{0\leq\tau\leq t}\{\lambda(\tau)\|E(\tau)\|_2\} \quad \text{(A8)}$$

Proof: From (5), (7) and (18), it is derived that

$$e(t)=y(t)-\{[1-\hat{A}(t,z^{-1})]y(t)+\hat{B}(t,z^{-1})u(t)\}$$

$$=\hat{A}(t,z^{-1})y(t)-\hat{B}(t,z^{-1})u(t) \quad \text{(A9)}$$

From (24) and the definition of $\varepsilon(t)$, we have

$$\hat{H}(t,z^{-1})u(t) = \hat{G}(t,z^{-1})\varepsilon(t)$$
$$= \hat{G}(t,z^{-1})y^*(t) - \hat{G}(t,z^{-1})y(t) \tag{A10}$$

Combining (A9) with (A10) yields the following equations

$$\left[\hat{A}(t,z^{-1})\hat{H}(t,z^{-1}) + \hat{B}(t,z^{-1})\hat{G}(t,z^{-1})\right]y(t) = T(z^{-1})y(t)$$
$$= \hat{H}(t,z^{-1})e(t) + \hat{B}(t,z^{-1}) \bullet \hat{G}(t,z^{-1})y^*(t)$$
$$+ \left[\hat{A}(t,z^{-1})\hat{H}(t,z^{-1}) - \hat{H}(t,z^{-1}) \bullet \hat{A}(t,z^{-1})\right]y(t)$$
$$+ \left[\hat{B}(t,z^{-1})\hat{G}(t,z^{-1}) - \hat{B}(t,z^{-1}) \bullet \hat{G}(t,z^{-1})\right]y(t)$$
$$+ \left[\hat{H}(t,z^{-1}) \bullet \hat{B}(t,z^{-1}) - \hat{B}(t,z^{-1}) \bullet \hat{H}(t,z^{-1})\right]u(t) \tag{A11}$$

$$\left[\hat{A}(t,z^{-1})\hat{H}(t,z^{-1}) + \hat{B}(t,z^{-1})\hat{G}(t,z^{-1})\right]u(t) = T(z^{-1})u(t)$$
$$= -\hat{G}(t,z^{-1}) \bullet e(t) + \hat{A}(t,z^{-1}) \bullet \hat{G}(t,z^{-1})y^*(t)$$
$$+ \left[\hat{B}(t,z^{-1})\hat{G}(t,z^{-1}) - \hat{G}(t,z^{-1}) \bullet \hat{B}(t,z^{-1})\right]u(t)$$
$$+ \left[\hat{A}(t,z^{-1})\hat{H}(t,z^{-1}) - \hat{A}(t,z^{-1}) \bullet \hat{H}(t,z^{-1})\right]u(t)$$
$$+ \left[\hat{G}(t,z^{-1}) \bullet \hat{A}(t,z^{-1}) - \hat{A}(t,z^{-1}) \bullet \hat{G}(t,z^{-1})\right]y(t)$$
$$\tag{A12}$$

From *Lemma 1*, it is clear that all the polynomials $\hat{A}(t,z^{-1})$, $\hat{B}(t,z^{-1})$, $\hat{H}(t,z^{-1})$ and $\hat{G}(t,z^{-1})$ have bounded coefficients.

Let us left-multiple $T^{-1}(z^{-1})$ on both sides of (A11) - (A12). Then based on the fact that $T(z^{-1})$ is a stable polynomial, we conclude that there exist positive constants $K_3^{'}$, $K_4^{'}$, $K_5^{'}$, $K_6^{'}$, $K_3^{''}$, $K_4^{''}$, $K_5^{''}$, $K_6^{''}$ such that

$$|y(t)| \le K_3^{'} + K_4^{'}\max_{0\le\tau\le t}\|E(\tau)\|_2 + K_5^{'}\max_{0\le\tau\le t}\|\Phi(\tau)\|_2 + K_6^{'}\max_{0\le\tau\le t}|y^*(\tau)| \tag{A13}$$

$$|u(t)| \le K_3^{''} + K_4^{''}\max_{0\le\tau\le t}\|E(\tau)\|_2$$
$$+ K_5^{''}\max_{0\le\tau\le t}\|\Phi(\tau)\|_2 + K_6^{''}\max_{0\le\tau\le t}|y^*(\tau)| \tag{A14}$$

It is obvious that $y^*(t)$ is bounded. Then further combining the above relations with the definition of $\Phi(t)$ yields that there exist positive constants K_3, K_4, K_5 such that

$$\max_{0\le\tau\le t}\|\Phi(\tau)\|_2 \le K_3 + K_4\max_{0\le\tau\le t}\|E(\tau)\|_2 + K_5\max_{0\le\tau\le t}\|\Phi(\tau)\|_2 \tag{A15}$$

Let us denote $\bar{K}_5 = 1$. Then for any $0 < K_5 < \bar{K}_5$, it is clear that there exist positive constants K_7, K_8 such that

$$\max_{0\le\tau\le t}\|\Phi(\tau)\|_2 \le \frac{K_3}{1-K_5} + \frac{K_4}{1-K_5}\max_{0\le\tau\le t}\|E(\tau)\|_2$$
$$= K_7 + K_8\max_{0\le\tau\le t}\|E(\tau)\|_2 \quad \text{(A16)}$$

From (20), it is known that $\|E(t)\|_2 - \sqrt{p}\Delta \le \lambda(t)\|E(t)\|_2$. By using this property, we have

$$\max_{0\le\tau\le t}\|\Phi(\tau)\|_2 \le K_7 + K_8\max_{0\le\tau\le t}\{\|E(\tau)\|_2 - \sqrt{p}\Delta + \sqrt{p}\Delta\}$$
$$\le K_7 + K_8\max_{0\le\tau\le t}\{\lambda(\tau)\|E(\tau)\|_2\} + K_8\sqrt{p}\Delta \quad \text{(A17)}$$

From (A17), it is known that there exist positive constants K_1, K_2 such that

$$\max_{0\le\tau\le t}\|\Phi(\tau)\|_2 \le K_1 + K_2\max_{0\le\tau\le t}\{\lambda(\tau)\|E(\tau)\|_2\} \quad \text{(A18)}$$

This proves *Lemma 2*.

Theorem 1: Let the nonlinear system (1) satisfy *Assumption* 1. For the closed-loop system (3) with the proposed controller, the following conclusions are obtained.

(i) The closed-loop system is stable in the sense that all the signals are globally bounded. Meanwhile, there exists an upper bound $\bar{\Delta}$ such that the output tracking error satisfies

$$\lim_{t\to\infty}|y(t) - y^*(t)| \le \bar{\Delta} \quad \text{(A19)}$$

(ii) Moreover, if at the steady state, the unmodeled dynamics $\zeta(t)$ and the reference $y^*(t)$ are slowly varying with respect to the sampling frequency such that (Chen, 2006)

$$\zeta(t) \approx \zeta(t-1) \approx \ldots \approx \zeta(t-N) \quad \text{(A20)}$$

$$y^*(t) \approx y^*(t-1) \approx \ldots \approx y^*(t-N) \quad \text{(A21)}$$

can hold for a large integer N, then the output tracking error satisfies

$$\lim_{t\to\infty}|y(t) - y^*(t)| \approx 0 \quad \text{(A22)}$$

Proof: (i) Using (22), (29) and the “key technical Lemma” in Goodwin and Sin (1984), it is concluded that: 1) $\{\lambda(t)\|E(t)\|_2\}_{t=1,2,\ldots}$ is a bounded sequence and 2) $\lim_{t\to\infty}\lambda(t)\|E(t)\|_2 = 0$.

Based on (A22), the first proposition denotes that: $\{\|\Phi(\tau)\|_2\}$ is a bounded sequence, all the signals must be bounded and the closed-loop system is stable.

From (20), the second proposition means that: $\lim_{t\to\infty}\|E(t)\|_2 \leq \sqrt{p}\Delta$ and $\lim_{t\to\infty}\lambda(t) = 0$, which shows that the recursive estimator gradually stops the online updating and can avoid continuous fluctuations.

Based on (A11), we also have

$$\lim_{t\to\infty}\left[\hat{H}(t,z^{-1}) \bullet \hat{A}(t,z^{-1}) + \hat{B}(t,z^{-1}) \bullet \hat{G}(t,z^{-1})\right]y(t) =$$

$$= \lim_{t\to\infty}\hat{B}(t,z^{-1}) \bullet \hat{G}(t,z^{-1})y^*(t) + \lim_{t\to\infty}\hat{H}(t,z^{-1})e(t)$$

$$+ \lim_{t\to\infty}\left[\hat{H}(t,z^{-1}) \bullet \hat{B}(t,z^{-1}) - \hat{B}(t,z^{-1}) \bullet \hat{H}(t,z^{-1})\right]u(t) \quad \text{(A23)}$$

From *Definition 1*, it is known that

$$\lim_{t\to\infty}\left[\hat{H}(t,z^{-1}) \bullet \hat{A}(t,z^{-1}) + \hat{B}(t,z^{-1}) \bullet \hat{G}(t,z^{-1})\right]$$

$$= \lim_{t\to\infty}\hat{B}(t,z^{-1}) \bullet \hat{G}(t,z^{-1}) \quad \text{(A24)}$$

and

$$\lim_{t\to\infty}\left[\hat{H}(t,z^{-1}) \bullet \hat{B}(t,z^{-1}) - \hat{B}(t,z^{-1}) \bullet \hat{H}(t,z^{-1})\right] = 0 \quad \text{(A25)}$$

Due to the conclusion $\lim_{t\to\infty}\|E(t)\|_2 \leq \sqrt{p}\Delta$, it is known that $\lim_{t\to\infty}\hat{H}(t,z^{-1})e(t)$ is also bounded.

Combining the above facts with the equation (A23) proves the conclusion (A19).

ii) Based on (3) and (18), the equation (A23) can be further extended to

$$\lim_{t\to\infty}\left[\hat{H}(t,z^{-1}) \bullet \hat{A}(t,z^{-1}) + \hat{B}(t,z^{-1}) \bullet \hat{G}(t,z^{-1})\right]y(t) =$$

$$= \lim_{t\to\infty}\hat{B}(t,z^{-1}) \bullet \hat{G}(t,z^{-1})y^*(t) + \lim_{t\to\infty}\hat{H}(t,z^{-1})\zeta(t)$$

$$+ \lim_{t\to\infty}\hat{H}(t,z^{-1})[\varphi^T(t)\theta] - \lim_{t\to\infty}\hat{H}(t,z^{-1})\left[\varphi^T(t)\hat{\theta}(t-1)\right]$$

$$+ \lim_{t\to\infty}\left[\hat{H}(t,z^{-1}) \bullet \hat{B}(t,z^{-1}) - \hat{B}(t,z^{-1}) \bullet \hat{H}(t,z^{-1})\right]u(t) \quad \text{(A26)}$$

If $\zeta(t)$ and $y^*(t)$ satisfy (A20) - (A21) at the steady state, then the system will be governed by the external input $u(t)$, which indicates that $\varphi(t)$ is also slowly varying

with respect to the sampling frequency, *i.e.*, $\varphi(t) \approx \varphi(t-1) \approx \ldots \approx \varphi(t-N)$ (Chen, 2006). It is obvious that there exists

$$\lim_{t\to\infty} \hat{H}(t, z^{-1})\zeta(t) \approx 0 \tag{A27}$$

$$\lim_{t\to\infty} \hat{H}(t, z^{-1})[\varphi^T(t)\theta] \approx 0 \tag{A28}$$

$$\lim_{t\to\infty} \hat{H}(t, z^{-1})\left[\varphi^T(t)\hat{\theta}(t-1)\right] \approx 0 \tag{A29}$$

Applying (A24), (A25), (A27), (A28) and (A29) to the equation (A26) can finally prove the conclusion (A22).
